# scMILD: Single-cell multiple instance learning for sample classification and associated subpopulation discovery

**DOI:** 10.1016/j.isci.2026.115284

**Published:** 2026-03-10

**Authors:** Kyeonghun Jeong, Jinwook Choi, Kwangsoo Kim

**Affiliations:** 1 Interdisciplinary Program in Bioengineering, Seoul National University, Seoul, Republic of Korea; 2 Institute of Medical and Biological Engineering, Medical Research Center, Seoul National University, Seoul, Republic of Korea; 3 Department of Transdisciplinary Medicine, Institute of Convergence Medicine with Innovative Technology, Seoul National University Hospital, Seoul, Republic of Korea; 4 Department of Medicine, College of Medicine, Seoul National University, Seoul, Republic of Korea

**Keywords:** Biocomputational method, Classification of bioinformatical subject, Transcriptomics

## Abstract

Linking cellular states to clinical phenotypes is a major challenge in single-cell analysis. Here, we present single-cell multiple instance learning for sample classification and associated subpopulation discovery (scMILD), a weakly supervised multiple instance learning framework that robustly identifies condition-associated cells using only sample-level labels. After systematically validating scMILD’s accuracy through controlled simulations, we applied it to diverse disease datasets, confirming its ability to retrieve known biological signatures. Building on this, our sample-informed analysis of scMILD-identified monocytes in COVID-19 revealed a temporal transition from an early antiviral to a late stress-response state. Furthermore, in a cross-disease application, a model trained on COVID-19 successfully stratified patients with Lupus and distinguished shared inflammatory states from disease-specific ones. scMILD thus provides a validated and versatile strategy to dissect cellular heterogeneity, bridging single-cell observations with high-level phenotypes.

## Highlights

• scMILD identifies condition-associated cells using only sample-level labels
• Dual-branch model with orthogonal projection loss improves cell separation
• Sample-informed analysis reveals monocyte state transitions in COVID-19 progression
• Cross-disease modeling reveals shared and specific signatures in SLE and COVID-19

## Graphical abstract

**Figure:**
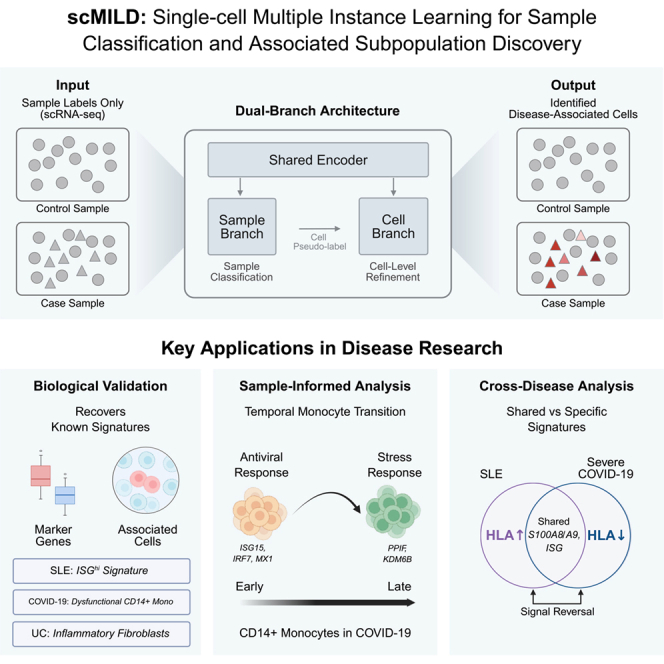

## Introduction

Single-cell transcriptomic data derived from various samples with diverse conditions and phenotypes have been accumulating rapidly.^1^^,^^2^^,^^3^ However, studies directly analyzing the association between sample conditions/phenotypes and individual cells still need to be completed. The primary analysis of single-cell RNA-seq often focuses on identifying condition/phenotype-specific or associated cell subsets.^4^^,^^5^^,^^6^ This process typically relies on unsupervised strategies, which can be time-consuming, require extensive knowledge of conditions/phenotypes, and involve subjective judgment from the analyst. These approaches also present challenges for reproducibility and validation, making it difficult to associate individual cells with sample conditions directly.

Recent computational advances have attempted to address these challenges. Deep learning approaches have enabled reference mapping with interpretable gene programs while improving clustering accuracy through various architectural innovations.^7^ Several methods have focused on improving cell type identification and clustering accuracy through advanced deep learning architectures, particularly addressing the challenges of rare cell populations.^8^^,^^9^ Other methods have leveraged bulk expression data to identify phenotype-associated subpopulations.^10^ While progress has been made in patient classification using single-cell data, current approaches focus on interpretability at the cell type level rather than identifying condition-specific cell states within each type.^11^ Moreover, these methods require extensive human intervention or additional data sources, limiting their practical application.

Multiple instance learning (MIL) offers a promising framework for addressing these challenges, having demonstrated success in various biological domains, including histopathological image analysis.^12^^,^^13^^,^^14^ In the context of single-cell transcriptomics, MIL’s bag-based learning paradigm naturally aligns with the hierarchical structure of single-cell data, where samples can be viewed as bags containing multiple cell instances. However, unlike other applications, single-cell transcriptomes present distinct challenges due to the need for ground truth labels at the cell level.

To address these limitations, we propose single-cell multiple instance learning for sample classification and associated subpopulation discovery (scMILD), a framework that leverages MIL to identify condition-associated cell subpopulations while providing quantitative performance metrics through sample-level classification. By treating samples as bags and cells as instances, scMILD offers a systematic approach to bridge sample-level phenotypes with cell-level molecular signatures.

To rigorously validate scMILD, we first demonstrate its technical robustness and the contribution of its key components through systematic simulations, ablation studies, and robustness tests against label noise. We further evaluate computational scalability and employ integrated gradient (IG) analysis to interpret the molecular features driving model predictions. We then benchmark its performance across diverse disease datasets. To showcase scMILD’s power to yield deeper biological insights, we present two distinct advanced applications. First, through a sample-informed analysis of COVID-19 patient data, we reveal dynamic temporal transitions in monocyte states during disease progression. Second, in a cross-disease framework, we use a model trained on COVID-19 to dissect cellular heterogeneity in Lupus. Together, these applications demonstrate that scMILD is a versatile tool capable of not only identifying disease-relevant cell populations but also exploring their dynamic changes over time and uncovering shared pathogenic mechanisms across different human diseases.

## Results

### Model architecture and training strategy

The scMILD framework is designed to address the “needle in a haystack” challenge inherent in single-cell analysis, which is governed by two biological assumptions: (1) a sample from a positive condition contains at least one positive-associated cell and (2) the majority of cells within that positive sample are not associated with the condition. This inherent sparsity means that if these few positive-associated cells can be accurately distinguished from the non-associated background, then assigning meaningful scores and classifying the entire sample becomes a much more direct and robust task. Consequently, scMILD operates on a central modeling premise: that accurate sample classification is best achieved as a consequence of learning a well-structured latent space where positive-associated and non-associated cells are geometrically separable.

This approach addresses a key limitation of standard MIL frameworks such as attention-based MIL model (ABMIL), whose training objective is solely focused on sample-level classification. As a result, a high attention score only signifies importance of an instance for the final prediction, not necessarily its association with the positive condition. For example, a cell with strong “negative” features in a negative sample could receive a high attention score because it helps the model confidently classify the sample as negative. This inherent ambiguity not only complicates the interpretation of attention scores but also means the model has no explicit incentive to organize different cell states within its latent space, failing to guarantee a structured representation suitable for downstream analysis.

**Figure 1:**
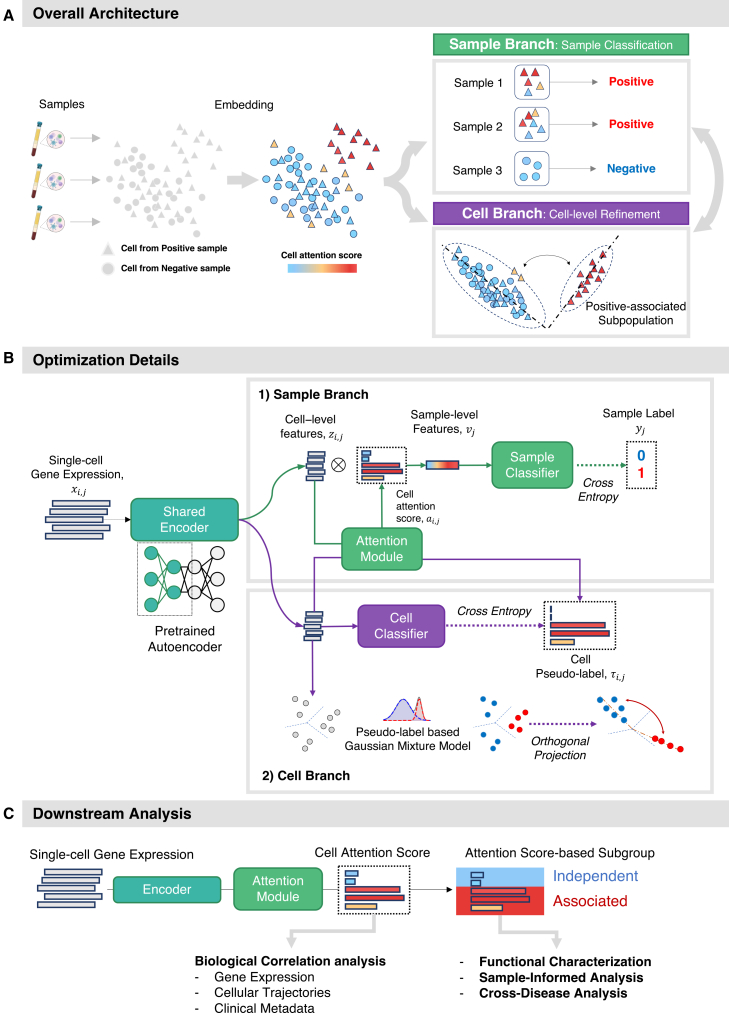
Overview of the scMILD framework (A) The scMILD model architecture and training process. The sample branch performs initial learning using sample-level classification to establish the model foundation, while the cell branch refines the model using cell-level prediction with pseudo-labels derived from attention scores. (B) Optimization details of the sample and cell branches. (C) Downstream analysis workflow utilizes cell attention scores for biological correlation analysis, functional characterization, sample-informed analysis and cross-disease analysis.

To resolve this ambiguity and actively structure the latent space, scMILD introduces a dual-branch architecture centered around a shared Encoder (Figure 1A). This architecture synergistically pursues the macroscopic goal of sample classification and the microscopic goal of refining cellular embeddings. The sample branch provides the initial directional signal by using sample-level labels and an attention mechanism to identify potentially positive-associated cells, generating attention scores that serve as pseudo-labels. Guided by these pseudo-labels, the cell branch then actively reshapes the latent space. Specifically, it employs a Gaussian mixture model (GMM) and an orthogonal projection loss (OPL) to enforce separation, pulling the embeddings of cells with high pseudo-labels closer together while ensuring they are orthogonal to those of cells with low pseudo-labels.

Through an alternating training process, these two branches cyclically update the shared Encoder (Figure 1B). This synergy ensures that scMILD constructs a well-structured latent space that not only yields high sample classification performance but also provides interpretable and reliable cell-level insights, effectively bridging cellular heterogeneity with high-level sample phenotypes.

### Simulation study results

**Figure 2:**
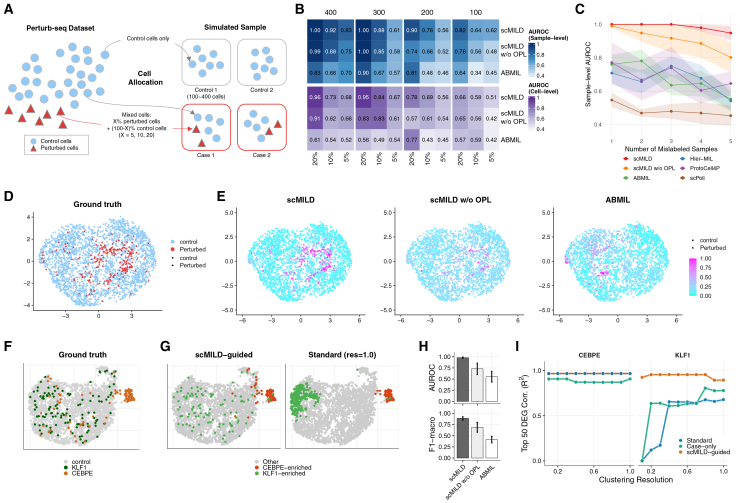
Performance evaluation of scMILD using simulations with single and mixed perturbation types (A) Schematic of simulated dataset construction from Perturb-seq data, with control (blue circles) and perturbed cells (red triangles) allocated to control and case samples. (B) Heatmap compares sample and cell classification AUROC for scMILD, scMILD w/o OPL, and ABMIL. Performance is evaluated across varying total cell counts per sample and proportions of perturbed cells in case samples. (C) Robustness to label noise. Performance (sample-level AUROC) as the number of mislabeled training samples increases (simulated dataset: 400 cells per sample, 20% perturbation, 14 training samples total). (D and E) UMAP visualizations of the median-performing test set show ground truth cell labels (D) and cell attention scores from each model (E), with perturbed cells represented as triangles and control cells as circles. (F) UMAP visualization of the mixture simulation dataset, colored by ground truth cell identity (control, KLF1-perturbed, CEBPE-perturbed). (G–I) show results from this mixture simulation. (G) UMAPs display clustering results from scMILD-guided clustering and standard clustering (resolution 1.0). (H) Bar plot compares sample classification performance (AUROC and F1-macro score) of the three models on the mixture simulation dataset. (I) Coefficient of determination $\left(r^{2}\right)$ from Pearson’s correlation between the log2 fold-change (log2FC) values of cluster-derived differentially expressed genes (DEGs) and those of the top 50 ground truth DEGs. For each method, the log2FCs from each identified cluster were compared against the ground truth, and the maximum $r^{2}$ value achieved is plotted. In (C), shaded areas represent standard error across 8 independent runs. In (H), bar heights represent the mean and error bars represent standard error of the mean (SEM) across 8 independent runs.

To objectively evaluate scMILD’s performance and practical utility in biological research, controlled simulation studies where ground truth cell labels were available through CRISPR-mediated perturbation were designed (Figure 2A). The model’s core capabilities were first assessed in a single-perturbation setting before testing its robustness in a more complex mixture simulation.

#### Performance in single-perturbation simulations

In the single-perturbation setting, analysis of sample classification performance revealed the superior robustness of scMILD across different experimental conditions. To specifically assess the impact of our proposed OPL, the full scMILD model was compared with an ablated version (scMILD w/o OPL) and a standard ABMIL. With 400 cells and 20% perturbation, scMILD achieved perfect sample classification (AUROC = 1.0), a notable improvement over scMILD w/o OPL (AUROC = 0.989) and ABMIL (AUROC = 0.833) (Figure 2B). Additional single-cell-based sample classification approaches were also evaluated.^11^^,^^15^^,^^16^ At the same experimental setting (400 cells, 20% perturbation), ProtoCell4P achieved reasonable performance (AUROC = 0.938) while Hier-MIL and scPoli showed limited effectiveness (AUROC = 0.500 and 0.469, respectively; Table S13). Cell-level performance showed similar trends, with scMILD achieving a cell AUROC of 0.956, compared to 0.905 for scMILD w/o OPL and 0.615 for ABMIL. Crucially, the strong correlation between sample and cell-level performance, particularly for the scMILD model, has a significant practical implication (Figure S3). Since true cell-level performance cannot be assessed in real-world datasets lacking ground-truth cell labels, a high, empirically measured sample AUROC can serve as a reliable proxy, providing confidence in the biological relevance of the underlying cell attention scores.

The model’s internal mechanism was then examined by analyzing the attention scores assigned to *KLF1*-perturbed and control cells. scMILD demonstrated a more apparent distinction between the two cell groups, achieving higher Kolmogorov-Smirnov statistics and lower overlap coefficients compared to the other models (Table S3; Figure S1). UMAP visualizations of the median-performing experiment confirmed that scMILD’s cell attention scores (Figure 2E) aligned better with the ground truth cell labels (Figure 2D) than those of the other models. Additional visualizations for experiments with lower cell counts are provided in Figure S2.

To assess the robustness of scMILD against annotation errors that commonly occur in clinical datasets, model performance was evaluated under increasing levels of label noise (Figure 2C). When training samples were progressively mislabeled (up to 5 mislabeled samples out of 14), scMILD maintained high performance (AUROC = 0.948), while scMILD w/o OPL showed substantial degradation (AUROC = 0.802). The additional benchmarked models showed even more severe performance decline, with ABMIL, ProtoCell4P, Hier-MIL, and scPoli achieving AUROCs of 0.552, 0.646, 0.542 and 0.453, respectively (Table S14). This demonstrates that the OPL not only improves baseline performance but also provides critical robustness against label noise, a key advantage for real-world applications where sample annotations may be imperfect.

Finally, we evaluated the practical utility of the identified cell groups for downstream analysis. Using cell attention scores to guide differential expression analysis, scMILD identified substantially more true-positive differentially expressed genes (DEGs) with higher recall rates compared to a standard phenotype-based analysis, especially in scenarios with low cell counts where the standard method failed entirely (Table S4). This demonstrates scMILD’s ability to effectively capture biological signals even in challenging, low-cell-count datasets.

#### Robust identification of heterogeneous cell populations in mixture simulations

To evaluate scMILD’s performance in more complex scenarios, we extended our framework to a mixture simulation including two distinct perturbed cell types (*KLF1*- and *CEBPE*-perturbed) mixed with control cells (Figure 2F). In this challenging sample classification task, scMILD achieved a mean AUROC of 0.9792 and an F1-macro score of 0.8889, consistently demonstrating superior performance over the ablated version, scMILD w/o OPL (AUROC = 0.7292, F1-macro = 0.6872), and the baseline ABMIL (AUROC = 0.5521, F1-macro = 0.4118) (Figure 2H).

To further evaluate the utility of scMILD for downstream analysis, three clustering strategies were compared: scMILD-guided clustering, standard clustering of all cells, and clustering of only case-derived cells (case-only). A critical finding was the strong dependency on the resolution parameter exhibited by the standard and case-only approaches, contrasted with the remarkable stability of scMILD-guided clustering. While the standard methods’ performance varied dramatically with resolution, scMILD maintained consistently high clustering quality across a broad range of resolution values, as measured by adjusted Rand index and adjusted mutual information (Figure S4D). This stability is a key advantage in real-world applications where optimal parameters cannot be known *a priori*.

Visualization of the clustering results further illustrated these differences. The scMILD-guided approach effectively separated *KLF1*- and *CEBPE*-perturbed cells into two distinct clusters, which we denote as *KLF1*-enriched and *CEBPE*-enriched (Figure 2G). In contrast, standard and Case-only clustering methods either failed to separate one of the perturbed populations or fragmented them across multiple clusters depending on the chosen resolution (Figures S4A–S4C).

Finally, to assess the biological relevance of the identified clusters, the coefficient of determination $\left(r^{2}\right)$ was calculated from the Pearson’s correlation between the log2 fold-changes (log2FC) of cluster-based DEG analysis results and the log2FCs of the top 50 ground truth DEGs (Figure 2I). For *KLF1*-perturbed cells, the log2FC values from the scMILD-guided *KLF1*-enriched cluster showed a remarkably high correlation with the ground truth ($r^{2}$ = 0.9353), outperforming the best-matching clusters from both Standard ($r^{2}$ = 0.6322) and Case-only ($r^{2}$ = 0.7288) approaches. For *CEBPE*-perturbed cells, the scMILD-guided approach again achieved the highest correlation ($r^{2}$ = 0.7631), substantially outperforming both the Standard ($r^{2}$ = 0.6171) and Case-only ($r^{2}$ = 0.2587) methods. This robust performance was maintained when correlating against all ground-truth DEGs (Figure S4E). Notably, for the standard and case-only comparisons, we selected the best-matching cluster from multiple candidates—an ideal scenario impossible in real analysis without prior knowledge. These results confirm that scMILD provides a more robust framework, yielding biologically accurate cell groupings while simultaneously reducing parameter sensitivity and eliminating subjective post-clustering decisions (Table S5).

### Performance evaluation on disease datasets

To evaluate scMILD’s effectiveness on real-world data, we conducted comprehensive benchmarking against multiple state-of-the-art models. Our comparison included an ablated version of our model (scMILD w/o OPL) to assess the contribution of key components, along with established MIL methods (ABMIL,^12^ Hier-MIL^15^) and recent single-cell-based sample classification approaches (ProtoCell4P,^11^ scPoli^16^). For a fair comparison between scMILD variants and ABMIL, we employed identical pre-trained autoencoder architectures, ensuring that performance differences reflect architectural innovations rather than feature extraction capabilities.

Performance was assessed using AUROC and F1-macro scores across four disease datasets (Table 1). scMILD achieved the highest AUROC scores in three of four datasets, with particularly strong performance in Lupus (1.0000) and UC (0.9750). In the COVID-19 Infection dataset (nasal swab) and COVID-19 hospitalization dataset (PBMC), scMILD achieved AUROCs of 0.9063 and 0.9562, respectively, consistently outperforming alternative approaches.

**Table 1:** Performance comparison of scMILD with other models on disease datasets

Metric	Dataset	scMILD	scMILD w/o OPL	ABMIL	ProtoCell4P	Hier-MIL	scPoli
AUROC	Lupus	**1.0000**	0.9997	0.9968	**1.0000**	0.9929	0.5522
	COVID-19 Infection	**0.9063**	0.8854	0.8646	0.8493	0.7188	0.5955
	COVID-19 Hosp.	**0.9562**	0.9529	0.9555	0.9549	0.8006	0.4945
	UC	**0.9750**	0.9583	0.8917	0.9370	0.9667	0.6167
F1-macro	Lupus	**0.9801**	0.9765	0.9635	0.9742	0.9163	0.5299
	COVID-19 Infection	**0.7769**	0.7562	0.6620	0.7097	0.6721	0.5823
	COVID-19 Hosp.	**0.9020**	0.8880	0.8957	0.8754	0.7020	0.4750
	UC	0.9001	0.8509	0.8514	0.5941	**0.9006**	0.6006

Bold values indicate the best performance.

The comparative analysis revealed several key insights. First, the stepwise performance improvement from ABMIL to scMILD w/o OPL to the full scMILD model validates our architectural innovations. The dual-branch structure (scMILD w/o OPL) improved upon ABMIL across all datasets, while the addition of OPL further enhanced performance, particularly evident in the UC dataset (AUROC: 0.9750 vs. 0.9583 for scMILD w/o OPL vs. 0.8917 for ABMIL).

Among the additional benchmarked models, ProtoCell4P showed competitive performance in Lupus (AUROC: 1.0000) but demonstrated lower performance in other disease contexts. Hier-MIL achieved reasonable performance in UC (AUROC: 0.9667) but struggled with COVID-19 datasets. Notably, scPoli exhibited limited success across all datasets, with AUROCs ranging from 0.4945 to 0.6167, suggesting challenges in adapting its multi-scale approach to these specific disease contexts.

The F1-macro scores (Table 1, bottom panel) corroborated these findings, with scMILD achieving the highest scores in three datasets. The only exception was UC, where Hier-MIL achieved a marginally higher F1-macro than scMILD (0.9006 vs. 0.9001), though scMILD maintained superior AUROC.

These results demonstrate that scMILD’s integrated approach—combining dual-branch architecture with GMM-based cell stratification and OPL—provides robust performance across diverse pathological contexts. The consistent superiority over established methods validates our framework for bridging sample-level phenotypes with cellular heterogeneity. Detailed performance metrics across all experimental runs are provided in Table S1.

### Computational efficiency and scalability

We assessed the computational efficiency and scalability of scMILD and baseline models using two dedicated benchmarks based on the Lupus dataset (Figure S5; Tables S15 and S16). To ensure a fair comparison of computational load, all benchmarks were run for a fixed 100 epochs without early stopping, using 3,000 HVGs as input.

First, in the cell-scaling experiments (Figures S5A and S5B; Table S15), we observed clear computational trade-offs. Hier-MIL was consistently the fastest model (154 s at 800k cells) but was also the most memory-intensive, with peak GPU usage scaling sharply to 18.0 GB. The scMILD variants (scMILD, scMILD w/o OPL, ABMIL) showed balanced, near-linear time scaling (e.g., scMILD: 6,718 s) and moderate memory usage (approx. 12.3 GB at 800k cells). Conversely, scPoli was the slowest (7,257 s) but demonstrated an exceptionally low memory footprint (approx. 50 MB), which likely reflects CPU-based operations rather than actual GPU utilization.

Next, in the sample-scaling experiments (Figures S5C and S5D; Table S16), a clear jump in both time and memory was observed for all models between the 20-sample (approx. 54k training cells) and 50-sample (approx. 96k training cells) data points. This is attributed to the smaller total cell count in the 20-sample dataset. Crucially, in the 50- to 150-sample range where training cell counts stabilized (95k-105k), both running time and peak GPU memory remained largely constant for all tested models. This finding demonstrates that for these MIL frameworks, the primary computational bottleneck is the “total number of cells,” not the number of samples (bags) into which they are organized.

### Validation of scMILD-identified condition-associated cell subpopulations

To validate the effectiveness of scMILD in identifying condition-associated cell subpopulations, the model was applied to four distinct datasets representing different disease conditions: Lupus, COVID-19 infection, COVID-19 hospitalization, and ulcerative colitis. For each dataset, we conducted downstream analysis on the median-performed test set. Our approach involved applying a 2-component GMM based on cell attention scores to divide cells derived from positive condition samples into two subgroups. The subgroup with higher scores was designated as the condition-associated subgroup, while the other was termed the condition-independent subgroup.

Analysis then focused on identifying and characterizing the condition-associated subgroup, comparing it with known subtypes or markers reported in previous studies. This systematic approach allowed us to evaluate scMILD’s performance across diverse pathological contexts and validate our findings against existing knowledge. Our analysis demonstrates the model’s capability to detect biologically relevant cell subtypes across various disease conditions, as detailed in the following sections.

#### Lupus: identification of SLE-associated cell subtypes

**Figure 3:**
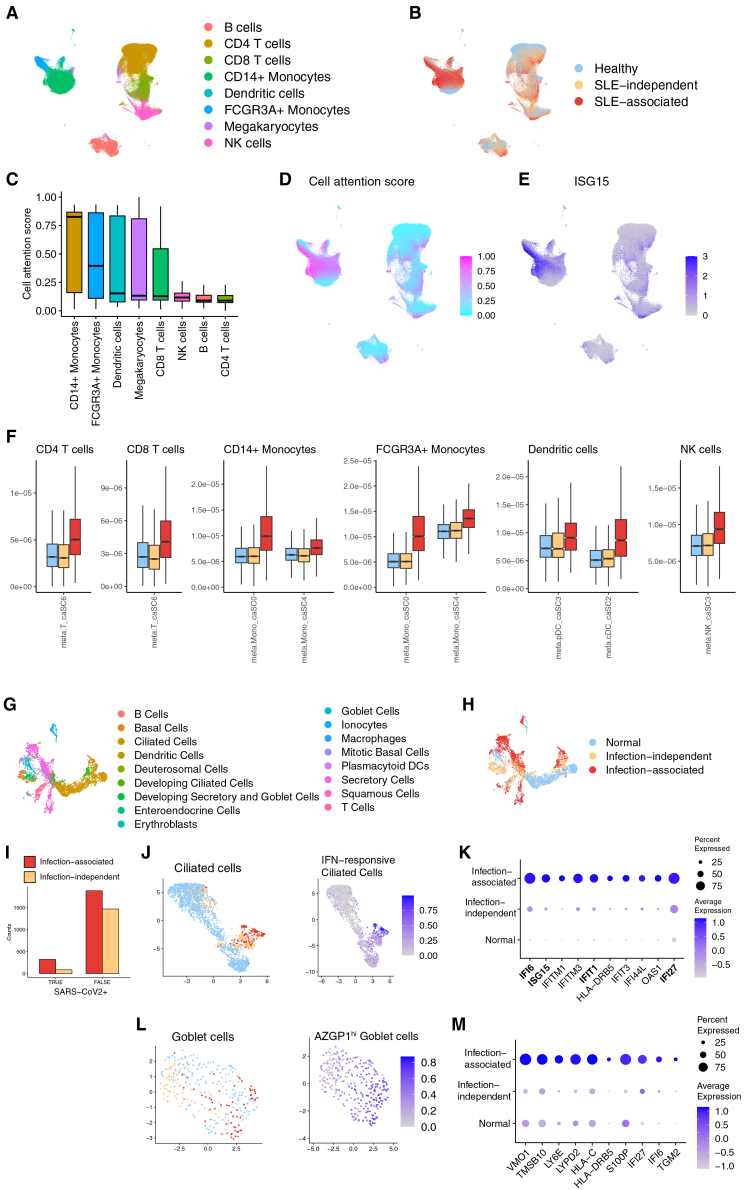
scMILD identifies disease-associated cell subpopulations in Lupus and COVID-19 infection datasets (A) UMAP visualization of the Lupus dataset, colored by cell type. (B) UMAP visualization of the Lupus dataset, colored by SLE association subgroup (healthy, SLE-independent, and SLE-associated). (C) Boxplot shows the distribution of cell attention scores for each cell type in the Lupus dataset. (D) Feature plot of cell attention scores in the Lupus dataset. (E) Feature plot shows the expression of ISG15 across cells in the Lupus dataset. (F) Boxplots compare meta-feature (ISG-high SLE-expanded subcluster marker gene) expression scores across healthy, SLE-independent, and SLE-associated subgroups for different cell types in the Lupus dataset. (G) UMAP visualization of the COVID-19 infection dataset, colored by cell type. (H) UMAP visualization of the COVID-19 infection dataset, colored by infection association subgroup (normal, infection-independent, and infection-associated). (I) Bar plot shows the proportion of SARS-CoV2 mRNA-detected cells in each subgroup. (J) UMAP plot of infection association subgroup and interferon-responsive ciliated cell subtype signature scores for ciliated cells. (K) Dot plot of top 10 differentially expressed genes in the infection-associated subgroup of ciliated cells. (L) UMAP plot of infection association subgroup and AZGP1-high Goblet cell subtype signature scores. (M) Dot plot of top 10 differentially expressed genes in the infection-associated subgroup of Goblet cells. In all boxplots, the center line represents the median, box edges represent the 25th and 75th percentiles, and whiskers extend to 1.5× the interquartile range.

The Lupus dataset comprised 50 samples from healthy individuals and 119 samples from patients with systemic lupus erythematosus (SLE). Figures 3A and 3B present UMAP visualizations of the dataset, providing insights into cellular heterogeneity and SLE-associated changes. In Figure 3A, we observe distinct clusters corresponding to different cell types, with notable populations of B, T, and monocytes. Figure 3B reveals the distribution of healthy, SLE-independent, and SLE-associated subgroups within these cell type clusters. Interestingly, the SLE-associated cells are not confined to specific regions but are distributed across multiple cell types, suggesting that SLE-associated changes occur within various cell populations.

Our analysis revealed that CD14^+^ monocytes and FCGR3A + monocytes exhibited the highest cell attention scores, consistent with previous research findings on SLE-associated cell types (Figure 3C). This observation aligns with the known involvement of monocytes in SLE pathogenesis.

To further investigate cellular heterogeneity, GMM was used on cell attention scores, independent of cell type, to divide cells from patients with SLE into “SLE-associated” and “SLE-independent” subgroups. Findings were then compared with previously reported SLE-associated cell subtypes.

A marker gene expression score was created by leveraging the 100 marker genes for each ISG^hi^ SLE-expanded subcluster reported in a previous study.^17^ Notably, the *ISG15* gene, common to all subclusters, showed a high Pearson’s correlation coefficient of 0.5997 with our cell attention scores (Figure 3D). The feature plot in Figure 3E further illustrates the expression pattern of *ISG15* across cells in the UMAP space, clearly showing higher expression in regions corresponding to the SLE-associated subgroup.

Furthermore, the expression of meta-features (top 100 marker genes of ISG^hi^ SLE-expanded subcluster) was compared across Healthy, SLE-independent, and SLE-associated subgroups for different cell types (Figure 3F). The top 100 DEGs for each ISG^hi^ SLE-expanded subcluster used in this analysis are listed in Table S6. The boxplots reveal a consistent pattern across all examined cell types: The SLE-associated subgroup demonstrates significantly higher expression of these marker genes than the healthy and SLE-independent subgroups. This trend is observed across various cell types, including monocytes, dendritic cells, and lymphocytes.

These results validate scMILD’s ability to identify disease-relevant cell subpopulations in SLE, aligning with and extending previous findings in the field. The consistency of our results across different cell types and the clear distinction in meta-feature expression between subgroups underscore the robustness of our approach in capturing disease-associated cellular states.

#### COVID-19 infection: detection of virus-specific cellular responses

The COVID-19 infection dataset, which includes measurements of SARS-CoV2 mRNA expression, provided insights into cellular heterogeneity and infection-associated changes. Figures 3G and 3H present UMAP visualizations of the dataset, revealing distinct cell type clusters and the distribution of normal, infection-independent, and infection-associated subgroups within these clusters.

The infection-associated cells were distributed across multiple cell types rather than forming a separate cluster. These cells tended to be located toward the periphery of their respective cell type clusters, suggesting that they represent states deviating from typical cellular profiles in normal conditions. Figure 3I quantitatively demonstrates the enrichment of SARS-CoV2 mRNA-detected cells in the infection-associated subgroup across all cell types, supporting the biological relevance of our identified subgroup (Table S8).

To validate our findings, results were compared with COVID-19 expanded cell subtypes reported previously.^18^ We focused on ciliated cells (1571:541, Normal:Infection) and Goblet cells (162:134, normal:infection), which had more than 100 cells in both normal and infection samples (Table S7).

For ciliated cells, we examined the distribution of the Interferon-responsive ciliated cell subtype, previously reported as expanded in patients with COVID-19. Using the top 5 DEG markers (*IFI6*, *ISG15*, *IFIT1*, *IFI27*, and *MX1*) for this known subtype, A strong correlation between the subtype signature score and our infection-associated subgroup was observed (Figure 3J). The Pearson’s correlation coefficient between the known subtype signature score and our cell attention score was remarkably high at 0.6348. Moreover, all five known subtype markers were present in the DEGs of our infection-associated subgroup, with four of them (excluding MX1) appearing in the top 10 DEGs (Figure 3K; Table S9). The dot plot in Figure 3K clearly shows the upregulation of these interferon-responsive genes in the infection-associated subgroup compared to the normal and infection-independent subgroups.

For Goblet cells, we analyzed the AZGP1^hi^ goblet cell subtype, another COVID-19 expanded cell subtype reported in the original study. Due to the very low *p*-values of the DEGs provided in the original paper, we selected the top 30 genes to calculate the signature score for this cell subtype. Similar to our findings in ciliated cells, we observed a strong association between this signature score and our infection-associated subgroup (Figure 3L). The Pearson’s correlation coefficient between the signature score and our cell attention score was high at 0.6564. Figure 3M shows the top 10 DEGs in the Infection-associated subgroup of Goblet cells, highlighting these cells’ distinct gene expression profiles in response to infection (Table S10). Notably, many of these genes, such as *LY6E*, *IFI6*, and *IFITM3*, are known to be involved in the interferon response, further confirming the biological relevance of our identified subgroups.

These results demonstrate scMILD’s ability to effectively identify virus-specific cellular responses in COVID-19 infection, corroborating previous studies while providing additional granularity in cell subtype identification.

#### COVID-19 hospitalization: characterization of severity-associated cell populations

**Figure 4:**
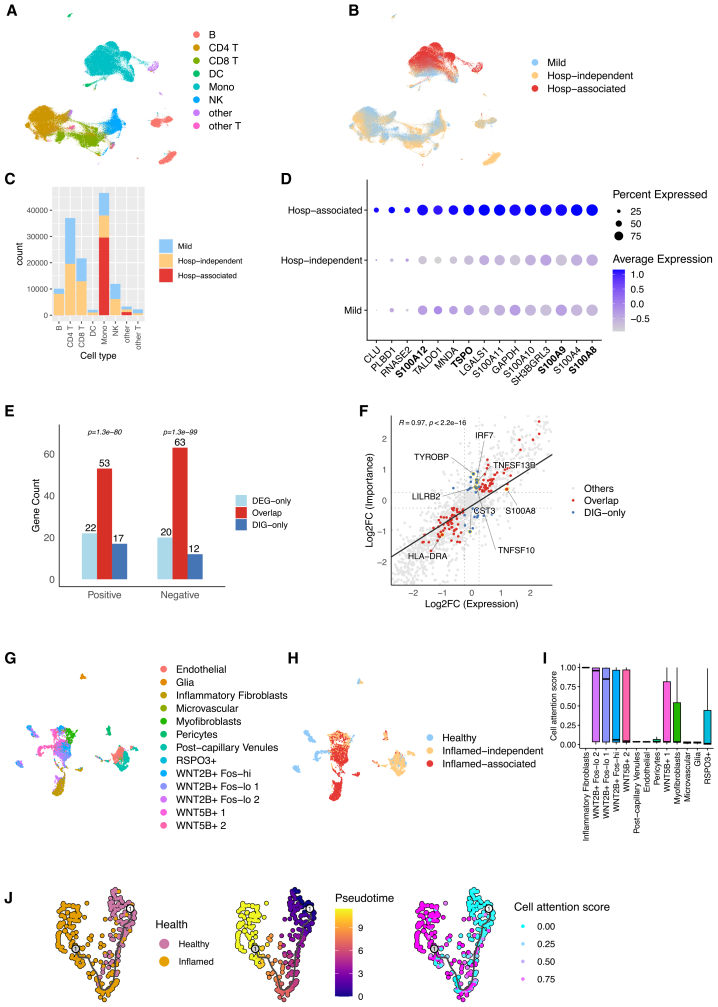
Characterization of scMILD-identified cell states in COVID-19 hospitalization and ulcerative colitis (A) UMAP visualization of the COVID-19 Hospitalization dataset, colored by cell type. (B) UMAP visualization of the dataset, colored by hospitalization association subgroup (mild, Hosp-independent, and Hosp-associated). (C) Stacked bar plot displays the number of cells in each subgroup for different cell types. (D) Dot plot shows the expression levels and percentage of cells expressing the top differentially expressed genes in the Hosp-associated CD14^+^ Monocytes subgroup compared to other subgroups. (E) Bar plot of DEGs and DIGs categorized into “overlap,” “DEG-only,” and “DIG-only” across positive and negative signatures. *p*-values indicate overlap significance (Fisher’s exact test, FDR-adjusted). (F) Scatterplot compares gene expression log2 fold-change (x axis) and model importance (y axis). Points are colored by “overlap,” “DIG-only,” and “others” categories. Dotted lines mark classification thresholds $\left(|\log_{2}\text{FC}|=0.25\right)$, and the solid line represents linear regression (R=0.97) with key driver genes annotated. (G and H) UMAP visualization of the UC test dataset colored by cell type (G) and subgroup (H). (I) Distribution of cell attention scores for each cell type according to sample condition in the UC dataset. (J) Trajectory analysis results for WNT2B+ Fos-lo 2 cells. Each column is colored by sample condition, pseudotime, and cell attention score. In all boxplots, the center line represents the median, box edges represent the 25th and 75th percentiles, and whiskers extend to 1.5× the interquartile range.

Cells were classified into Hosp-associated, Hosp-independent, and Mild subgroups in the COVID-19 Hospitalization PBMC dataset, comprising 254 COVID-19 PBMC samples. Figures 4A and 4B present UMAP visualizations of the dataset, revealing distinct cell type clusters and precise separation between the hospitalization association subgroups. The UMAP plots demonstrate that certain cell types, mainly monocytes, show a higher proportion of Hosp-associated cells, suggesting their potential role in disease severity.

Most cells in the Hosp-associated subgroup were identified as monocytes at the broad cell type level, with CD14^+^ monocytes being the predominant cell type at a more specific level. This is clearly illustrated in Figure 4C, where the stacked bar plot shows a higher proportion of Hosp-associated cells in the Mono (monocyte) category than other cell types.

Our analysis revealed a striking similarity between the Hosp-associated subgroup and the “dysfunctional CD14 monocytes” subtype reported in the original study.^4^ All marker genes for this subtype (*S100A8*, *S100A12*, *S100A9*, *S100A6*, and *TSPO*) were among the top 15 DEGs in our Hosp-associated subgroup within the CD14^+^ monocytes cell type (Table S11). Figure 4D presents a dot plot of these DEGs, where the size of the dots represents the percentage of cells expressing the gene and the color intensity indicates the average expression level. The plot clearly shows the upregulation of these marker genes in the Hosp-associated subgroup compared to the Hosp-independent and mild subgroups, particularly for genes such as *S100A8*, *TSPO*, *S100A9*, and *S100A12*.

To delve deeper into the molecular features driving scMILD’s predictions beyond these canonical markers, IGs were used to compute gene-level feature importance scores for CD14^+^ monocytes. The model-derived importance scores showed a high degree of concordance with biological expression differences (Spearman ρ=0.925, Pearson’s r=0.969; Figure 4F). Genes identified as both DEGs and differentially important genes (DIGs) included all marker genes of the dysfunctional CD14 monocyte subtype described above, as well as suppressed antigen presentation markers (e.g., *HLA-DPB1* and *HLA-DPA1*). This confirms that scMILD correctly prioritizes well-known pathogenic signatures associated with hospitalized COVID-19.

Beyond recapitulating expression differences, scMILD prioritized regulatory genes that were uniquely identified by IG analysis (DIG-only genes; Table S18). These genes exhibited high model importance despite showing modest fold changes that fell below the threshold for traditional DEG classification $\left(|\log_{2}\text{FC}|<0.25\right)$. A prime example is *IRF7*, the master regulator of type I interferon responses.^19^ While its expression change was modest $\left(\log_{2}\text{FC}=0.16\right)$, scMILD assigned it disproportionately high importance $\left(\log_{2}{\text{FC}}_{\text{IG}}=0.67\right)$. Similarly, *TYROBP* (DAP12), a critical signaling adaptor for myeloid activation,^20^ was prioritized as a top-tier driver $\left(\log_{2}{\text{FC}}_{\text{IG}}=0.86\right)$ despite negligible expression changes $\left(\log_{2}\text{FC}=0.05\right)$. This suggests that scMILD captures regulatory importance beyond transcriptional magnitude, recognizing key signaling hubs that drive the cellular state.

Furthermore, the model assigned high importance to *TNFSF10* (TRAIL) and *TNFSF13B* (BAFF), ligands involved in lymphocyte apoptosis and B-cell activation, respectively. Notably, elevated expression of these cytokines has been observed in immune cells from patients with COVID-19,^21^ and their high importance here implies that scMILD’s decision-making reflects the monocyte’s potential role in mediating systemic immunopathology. Additionally, the strong negative importance of *CST3*, a protease inhibitor $\left(\log_{2}{\text{FC}}_{\text{IG}}=-1.02\right)$, points to an impaired protease-antiprotease balance, consistent with the tissue-damaging inflammatory milieu observed in hospitalized cases.

Collectively, these results validate scMILD’s ability to identify severity-associated cell populations in COVID-19. Not only do the findings align closely with previous reports of dysfunctional monocytes, but the identification of upstream regulators and systemic effectors as key drivers highlights the potential of scMILD to uncover deeper, clinically relevant biological mechanisms in complex diseases.

#### Ulcerative colitis: identification of inflammation-specific fibroblasts

In the Ulcerative colitis dataset, analysis focused on inflammatory fibroblasts, a cell type-specific to the inflamed condition (Figures 4G and 4H). Our analysis confirmed that scMILD appropriately assigned high cell attention scores to this cell type, validating the model’s ability to recognize disease-specific cell populations (Figure 4I). Interestingly, the WNT2B+ Fos-lo 2 cell type exhibited the second-highest median cell attention score, with a wide distribution. Further investigation through pseudotime analysis revealed a strong correlation between pseudotime and cell attention scores within this cell type, with a Pearson’s correlation coefficient of 0.6 (Figure 4J). This correlation suggests that the cell attention scores assigned by scMILD may reflect these cells’ developmental or activation trajectory in the context of ulcerative colitis.

These findings corroborate the original study’s identification of inflammatory fibroblasts as a hallmark of inflamed tissue in ulcerative colitis and provide insights into the potential developmental trajectories of disease-associated cell types.

In summary, across these four diverse disease contexts, scMILD consistently demonstrated its ability to identify condition-associated cell subpopulations that align with and extend previous findings. Our analysis revealed several key strengths of the scMILD approach: 1. Robustness: scMILD effectively identified relevant cell subpopulations across various pathological conditions, from autoimmune diseases to viral infections and inflammatory disorders. 2. Biological relevance: The condition-associated subgroups identified by scMILD showed strong correlations with known disease-specific markers and subtypes reported in previous studies. 3. Additional insights: Besides corroborating existing knowledge, scMILD provided perspectives on cellular heterogeneity in disease contexts, such as the potential developmental trajectories of disease-associated cell types in ulcerative colitis. 4. Versatility: scMILD’s performance across different tissue types (e.g., PBMCs, nasal epithelium, and colon) demonstrates its adaptability to various biological systems. 5. Granularity: The model’s ability to identify condition-associated cells within specific cell types offers a more nuanced understanding of disease processes at the cellular level.

These results demonstrate scMILD’s robustness and potential for discovering insights into cellular heterogeneity in various pathological conditions. By leveraging cell attention scores to identify associated subpopulations, scMILD provides a powerful tool for uncovering the cellular basis of complex diseases, potentially leading to avenues for therapeutic interventions and personalized medicine approaches.

### Sample-informed/analysis of cellular states: A COVID-19 hospitalization case study

To investigate the functional heterogeneity within disease-relevant monocytes identified by scMILD, a sample-informed analytical approach focused on high-attention CD14^+^ monocytes from COVID-19 hospitalized patients was used. By generating pseudobulk expression profiles from these cells for each sample, we preserved both cellular transcriptional signatures and their sample-level context.

**Figure 5:**
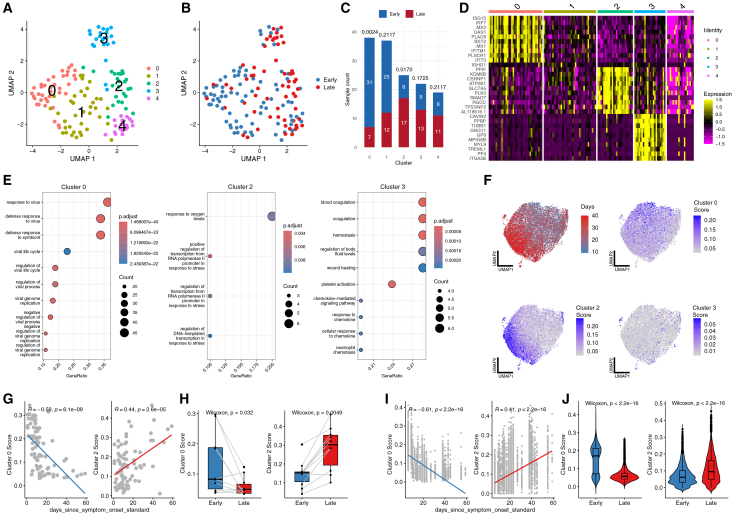
Sample-informed analysis reveals temporal monocyte dynamics in COVID-19 hospitalization (A) UMAP visualization of pseudobulk profiles from high-attention CD14 monocytes, colored by clustering results showing five distinct sample groups. (B) UMAP visualization of the same profiles colored by time points (early, late post-symptom onset). (C) Stacked bar plot shows the distribution of samples collected at early and late across each cluster with Fisher’s exact test *p*-values indicated. (D) Heatmap displays the top 10 differentially expressed genes for each pseudobulk cluster. (E) Dot plot of Gene Ontology enrichment analysis results for clusters 0, 2, and 3. (F) UMAP visualization of single cells from the external validation dataset, colored by days post-symptom onset and score distributions for clusters 0, 2, and 3. (G) Scatterplot illustrates the correlation between cluster scores and time since symptom onset at the pseudobulk sample level. (H) Paired boxplots compare cluster 0 and cluster 2 scores between early and late timepoints in longitudinal samples from the same patients. (I) Scatterplot shows the correlation between cluster scores and time since symptom onset at the single-cell level. (J) Violin plots compare the distribution of cluster 0 and cluster 2 scores between early and late cells. In all boxplots, the center line represents the median, box edges represent the 25th and 75th percentiles, and whiskers extend to 1.5× the interquartile range.

Unsupervised clustering of these pseudobulk profiles revealed five distinct sample groups (Figure 5A), with three clusters displaying characteristic functional signatures.

Differential gene expression analysis identified cluster-specific marker genes (Figure 5D). Cluster 0 exhibited elevated expression of interferon-stimulated genes (*ISG15*, *IRF7*, *MX2*, *IFITM1*, and *MX1*). Cluster 2 was defined by the upregulation of stress response genes (*PPIF*, *KDM6B*, *CSRNP1*, *ATP2B1*, and *PLK3*). Cluster 3 showed the upregulation of genes associated with platelet activation processes (*CAVIN2*, *PPBP*, *TUBB1*, *GP9*, *PF4*, and *ITGA2B*).

Gene Ontology enrichment analysis revealed distinct biological pathways associated with each major cluster (Figure 5E). Cluster 0 showed significant enrichment of antiviral response pathways. Cluster 2 was enriched for response to oxygen levels and stress-induced transcriptional regulation pathways. Cluster 3 demonstrated enrichment of platelet-related processes.

Integration of clinical metadata revealed distinct temporal associations in monocyte transcriptional states (Figure 5B). When samples were colored by time since symptom onset, a separation between early and late samples emerged on the UMAP projection. Quantitative analysis confirmed this temporal association (Figure 5C): The early antiviral response profile (cluster 0) predominated in samples collected early post-symptom onset (31/38 samples, Fisher’s exact test *p* = 0.0024), while the stress response profile (cluster 2) was significantly associated with samples collected late post-symptom onset (17/25 samples, Fisher’s exact test *p* = 0.0170).

Notably, the temporal dynamics of these transcriptional states partially align with previous observations in independent COVID-19 cohorts. The early interferon response signature we identified in cluster 0 includes established markers such as *ISG15*, which has been reported by^22^ to show highest expression at early time points with consistent decrease over time. For the late-stage stress response signature (cluster 2), prior research by^23^ reported *PPIF* as one of the only two genes showing increased expression at later disease stages, but failed to establish a significant gene set-level association with disease progression.

To validate the reproducibility of these temporal dynamics, we applied our analytical framework to an independent COVID-19 dataset with longitudinal sampling.^24^ At the pseudobulk level, our identified molecular signatures showed significant correlations with time since symptom onset (Figure 5G; cluster 0: r = −0.59; Cluster 2: r = 0.44). Paired analysis of longitudinal samples from the same patients further confirmed significant shifts in signature scores over time (Figure 5H; cluster 0 decreased: *p* = 0.032; cluster 2 increased: *p* = 0.0049).

Our sample-informed approach demonstrates that these temporal patterns are more robustly captured through gene signature scores than individual markers. The correlation between days since symptom onset and our gene signature scores (cluster 0: r = −0.61; cluster 2: r = 0.41) substantially exceeded the correlations observed with individual genes such as *ISG15* (r = −0.42) and *PPIF* (r = 0.18). This suggests that coordinated gene programs, rather than individual markers, better reflect the biological transitions occurring during disease progression.

These results demonstrate a programmatic transition in monocyte functionality during COVID-19 progression from an initial interferon-mediated antiviral state to a subsequent stress adaptation state characterized by response to oxygen levels and stress-induced transcriptional regulation. While previous studies have identified individual components of these responses,^22^^,^^23^ our sample-informed approach provides a comprehensive view of this cellular state transition at both population and single-cell levels, offering insights into the dynamic nature of immune responses during COVID-19 pathogenesis.

### Cross-disease application of scMILD reveals shared and specific cellular states in COVID-19 and SLE

To evaluate the cross-disease generalizability of scMILD, reciprocal predictions between COVID-19 and SLE were performed. The scMILD model trained on COVID-19 patient data predicted disease states in an independent Lupus cohort with high accuracy (AUROC 0.9012, F1-macro 0.8376). Conversely, the model trained on Lupus data showed significantly reduced performance when applied to patients with COVID-19 (AUROC 0.7176, F1-macro 0.7251).

**Figure 6:**
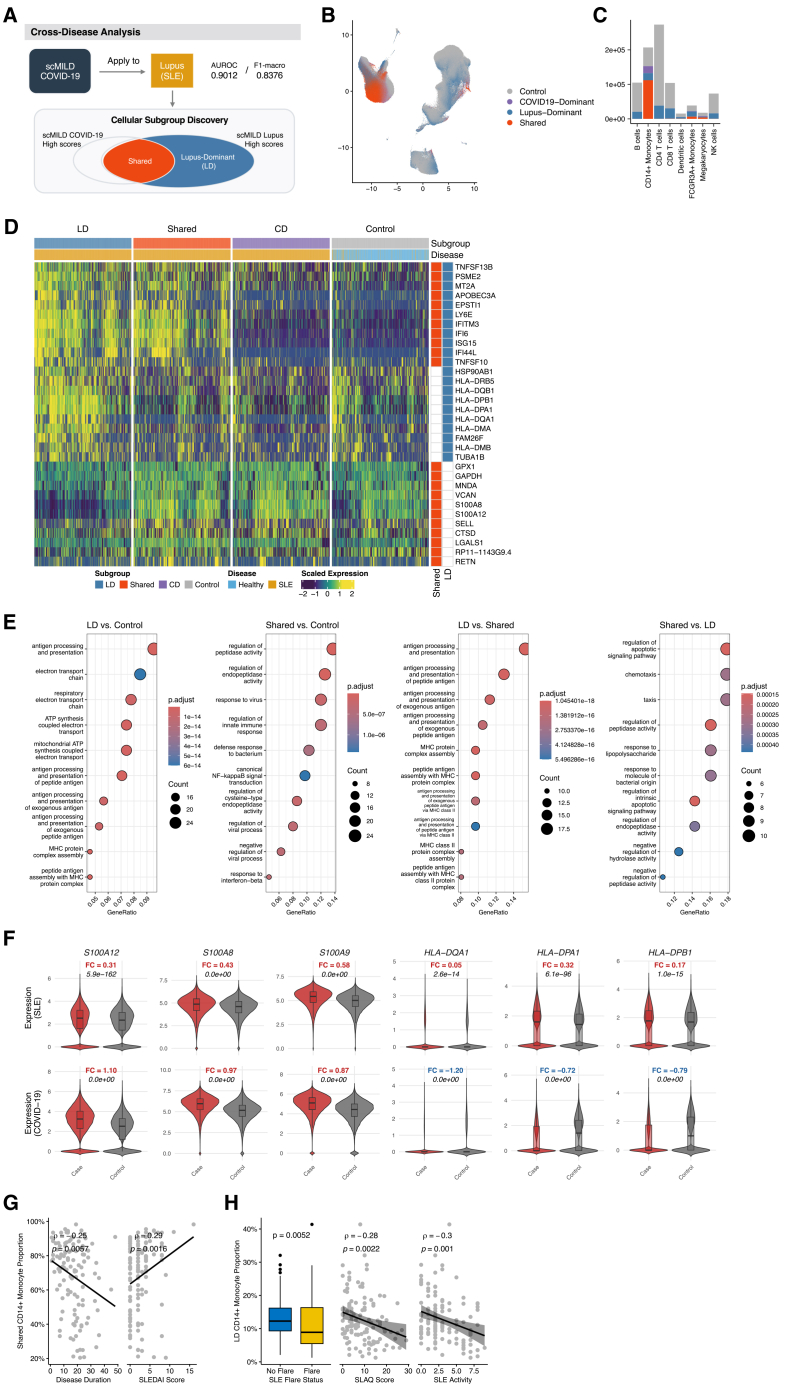
Cross-disease application of scMILD reveals shared and specific cellular states in COVID-19 and Lupus (A) Schematic of the cross-disease analysis workflow. An scMILD model trained on COVID-19 data is applied to the Lupus dataset. Cells are then categorized into shared, Lupus-dominant (LD), COVID19-dominant, and control subgroups based on their attention scores. (B) UMAP visualization of all cells from the Lupus dataset, colored by the cross-disease subgroups defined in (A). (C) Stacked bar plot shows the cellular composition of each cross-disease subgroup across major cell types. (D) Heatmap shows scaled expression of the union of top 10 DEGs from the shared vs. control and LD vs. control comparisons. The heatmap includes 1,000 subsampled cells per group, with rows annotated to highlight genes specific to Shared or LD signatures. (E) Dot plot displays the top 10 enriched Gene Ontology (GO) Biological Process terms for differentially expressed genes (DEGs) from comparisons between shared, Lupus-dominant (LD), and control CD14^+^ Monocytes. (F) Violin plots compare the expression distributions of representative genes for the “shared” (left; *S100A12, S100A8, S100A9*) and “Lupus-dominant (LD)” (right; *HLA-DQA1, HLA-DPA1,* and *HLA-DPB1*) signatures. The comparisons are stratified by disease status (case vs. control) across the Lupus (top row) and COVID-19 (bottom row) datasets. (G) Scatterplots show the correlation between the proportion of shared CD14^+^ monocytes and clinical variables, including disease duration and SLEDAI score. (H) Clinical associations for the proportion of Lupus-dominant (LD) CD14^+^ monocytes. A boxplot compares proportions by SLE flare status, and scatterplots show correlations with SLAQ score and overall SLE activity. In all boxplots, the center line represents the median, box edges represent the 25th and 75th percentiles, and whiskers extend to 1.5× the interquartile range.

To investigate the molecular basis of this asymmetry and explore single-cell heterogeneity within Lupus, cell-level scores from both the COVID-19-trained and Lupus-trained models were integrated. Cells were categorized into four groups based on their score concordance: “shared”’ (high scores from both models), “Lupus-dominant” (LD; high Lupus score, low COVID-19 score), “COVID19-dominant” (CD; high COVID-19 score, low Lupus score), and “control” (low scores from both models or healthy control cells) (Figure 6A). Distribution analysis across all cell types (Figures 6B and 6C) revealed “shared” cells were most prominent within CD14^+^ monocytes (54.1%), aligning with our previous findings of high scMILD scores in CD14^+^ monocytes from hospitalized patients with COVID-19 and suggesting their pivotal role in common pathogenic mechanisms. Subsequent analyses focused on the molecular features of “shared” and “LD” CD14^+^ monocyte subpopulations in SLE.

DEG analysis comparing “shared,” “LD,” and “control” CD14^+^ monocytes (Figures 6D and 6E) showed that interferon-response genes (*ISG15*, *IFI6*, and *IFITM3*) were commonly upregulated in both “shared” and “LD” groups versus “control” cells, indicating activated interferon-mediated inflammation. Direct comparison revealed distinct signatures: “LD” CD14^+^ monocytes had significantly higher expression of MHC class II-related genes (*HLA-DQA1* and *HLA-DPB1*), while “shared” CD14^+^ monocytes were characterized by high expression of previously reported dysfunctional monocyte markers from COVID-19 (*S100A8/A9/A12* and *TSPO*).

GO enrichment analysis showed “LD” CD14^+^ monocytes were primarily enriched for “antigen processing and presentation” GO biological process terms compared to both “control” and “shared” groups, suggesting enhanced antigen presentation capacity. “Shared” CD14^+^ monocytes, versus “control,” were enriched for “innate immune response,” “response to virus,” and “response to interferon-beta.” Compared to “LD” cells, “shared” cells showed enrichment for the “regulation of apoptotic signaling pathway” and “regulation of peptidase activity,” implying involvement in general inflammatory and specific stress responses.

Importantly, the “LD” group provided the mechanistic explanation for the limited generalizability of the SLE model. Comparing gene expression between SLE cases and severe COVID-19 cases revealed a critical “signal reversal” (Figure 6F). While MHC class II genes were significantly upregulated in SLE cases, they were downregulated in severe COVID-19 cases due to impaired antigen presentation. Consequently, the SLE-trained model, which learned to associate high HLA expression with the disease state, failed to recognize patients with severe COVID-19 exhibiting the low HLA phenotype, misclassifying them as controls.

Clinical correlation analysis (Figures 6G and 6H; Table S12) further validated these distinct immunological roles. The proportion of “shared” CD14^+^ monocytes positively correlated with the SLEDAI score (ρ=0.29, FDR < 0.05) and negatively with disease duration (ρ=−0.25, FDR < 0.05), associating this acute inflammatory signature with active clinical flares. Conversely, “LD” CD14^+^ monocytes were significantly more abundant in non-flare patients (Wilcoxon *p* = 0.0052) and negatively correlated with the Systemic Lupus Activity Questionnaire (SLAQ) score (ρ=−0.28, FDR < 0.05). This suggests that the “LD” signature reflects a chronic, adaptive immune state maintained during stable disease phases.

Synthesizing these findings reveals two key immunological components in SLE pathophysiology. First, “shared” CD14^+^ monocytes represent an “acute inflammatory module”—cells executing innate immune and viral defense programs activated during clinical flares. This molecular trajectory shares substantial similarity with COVID-19’s acute inflammatory response, explaining the COVID-19 model’s successful detection of active SLE. Second, “LD” CD14^+^ monocytes constitute a “chronic adaptive immune signature”—specialized in antigen presentation and maintaining the chronic immunological state characteristic of autoimmunity. The suppression of this signature in severe COVID-19 (signal reversal) explains why the SLE model fails to recognize COVID-19’s acute immune dysfunction.

Collectively, these results demonstrate that scMILD reveals distinct functional heterogeneity in SLE monocytes by defining two contrasting functional states. The first is an “acute inflammatory state” (“shared” subtype), characterized by S100A family alarmins and associated with high disease activity. The second is a “specialized antigen-presenting state” (“LD” subtype), defined by high MHC-II expression and associated with disease stability. While recent large-scale studies have identified similar monocyte subtypes in SLE at the single-cell level,^17^^,^^25^ our cross-disease modeling approach demonstrates that a model trained solely on COVID-19 data can successfully stratify these clinically relevant subpopulations in an independent autoimmune disease. Furthermore, although bulk transcriptome analyses have revealed shared interferon-driven signatures between severe COVID-19 and SLE,^26^ our study provides single-cell resolution of this cross-disease inflammatory axis. This molecular dichotomy offers a framework for understanding SLE heterogeneity and suggests that the “shared”/”LD” ratio may serve as a potential biomarker for patient stratification.

## Discussion

scMILD demonstrated robust performance across diverse single-cell RNA-seq datasets, highlighting its potential for broad application in various biological contexts. The model’s effectiveness stems from its dual-branch architecture, where the sample branch performs robust sample-level classification while the cell branch identifies condition-associated cell subpopulations through enhanced representation learning. Unlike conventional unsupervised clustering approaches that require extensive knowledge and subjective judgment, scMILD systematically leverages sample-level labels to identify condition-associated cellular populations, generally outperforming state-of-the-art models such as ABMIL, Hier-MIL, ProtoCell4P, and scPoli.

Our mixture simulation studies revealed a significant advantage of scMILD in addressing parameter sensitivity issues that plague standard clustering methods. While conventional approaches require extensive parameter tuning to optimally identify different cell types, scMILD maintained consistent high performance across a broad range of resolution values. This stability is particularly valuable in real-world applications where optimal parameters cannot be determined *a priori*. Furthermore, scMILD demonstrated superior ability to detect DEGs compared to phenotype-based analysis, particularly in datasets with limited cell numbers where conventional approaches failed completely.

The utility of scMILD in dissecting cellular heterogeneity is further exemplified by its application in complex disease scenarios. For instance, the sample-informed analysis in COVID-19 hospitalization revealed temporal monocyte transitions, with gene signatures robustly tracking disease progression. Moreover, the IG analysis demonstrated that scMILD’s predictions capture regulatory genes whose importance exceeds their transcriptional magnitude—such as the interferon master regulator *IRF7* and the myeloid signaling adaptor *TYROBP*—highlighting the model’s capacity to identify mechanistic drivers beyond differential expression.

Extending this, our cross-disease analysis between COVID-19 and Lupus demonstrated scMILD’s capacity to identify both shared inflammatory states and disease-specific cellular characteristics. The “shared” and “Lupus-dominant” monocyte subtypes we identified aligned with recent independent observations of ISG-high and MHC-II-high monocyte populations in Lupus, which showed opposing associations with disease activity. Critically, our approach achieved comparable biological insights using only sample-level labels through cross-disease modeling, providing single-cell resolution of the inflammatory axis shared between COVID-19 and Lupus. Crucially, scMILD linked these distinct monocytic states to different clinical trajectories, showcasing its power to define clinically relevant cellular heterogeneity across different pathological contexts.

This study presents scMILD as a significant contribution to single-cell transcriptomics by establishing a systematic, reproducible method for connecting sample conditions to specific cell subpopulations. CRISPR-perturbation simulation studies definitively demonstrated scMILD’s ability to accurately identify condition-associated cells in both single and mixed perturbation scenarios. The model’s excellent performance, even with limited cell numbers and reduced parameter sensitivity, enhances its practicality. Notably, across diverse disease datasets including Lupus, COVID-19, and ulcerative colitis, scMILD not only consistently outperformed state-of-the-art models but also identified condition-associated cell subpopulations that aligned with findings from original studies. These capabilities offer possibilities for understanding disease-relevant cellular states and monitoring disease progression, ultimately advancing our understanding of cellular heterogeneity and its role in disease mechanisms.

### Limitations of the study

Despite these strengths, several limitations should be acknowledged. First, the current implementation focuses on binary classification problems, requiring extension for more complex multi-class scenarios. Second, while our label noise experiments demonstrated that scMILD maintains robust performance even with up to 35% mislabeled training samples, the model’s predictions are ultimately bounded by the quality of sample-level annotations; systematic biases in labeling could still propagate to cell-level inferences. Third, while the IG analysis partially addresses interpretability by identifying key molecular drivers, a complete mechanistic understanding of the features driving cell-level attention scores warrants further investigation. Fourth, computational scalability presents trade-offs: scMILD demonstrates stable memory usage and near-linear time scaling, but does not achieve the fastest runtime nor the lowest memory footprint compared to specialized alternatives, reflecting the overhead of its dual-branch architecture.

Finally, the model identifies condition-associated cells regardless of their annotated type, a strength for discovering unannotated or cross-type cellular states. However, because scMILD optimizes for sample-level classification, the attention mechanism inherently prioritizes signals that are both strong and prevalent across samples. This design choice means that rare cell populations or subtle, cell-type-specific alterations—which may not substantially contribute to sample-level discrimination—could be underrepresented in the global attention distribution. We emphasize that this reflects an inherent trade-off of the weakly supervised MIL paradigm, where sample-level labels provide the only supervisory signal, rather than a failure of the method. To comprehensively assess cell-type-specific responses, we recommend dedicated post-hoc integration of scMILD outputs with cell type annotations. Future extensions incorporating hierarchical or cell-type-aware attention mechanisms could potentially address this limitation while maintaining the framework’s sample-level interpretability.

## Resource availability

### Lead contact

Requests for further information and resources should be directed to and will be fulfilled by the lead contact, Kwangsoo Kim (kwangsookim@snu.ac.kr).

### Materials availability

This study did not generate new materials.

### Data and code availability

• The publicly available datasets used in this study can be accessed from their original repositories. The Lupus dataset is available on GitHub (https://github.com/yelabucsf/lupus_1M_cells_clean). The COVID-19 infection dataset is available through the Single Cell Portal under accession code SCP1289. The COVID-19 Hospitalization dataset (Su et al.) can be accessed via the Fred Hutch COVID-19 Atlas (https://atlas.fredhutch.org/fredhutch/covid/dataset/su). The COVID-19 longitudinal PBMC dataset (Stephenson et al.) is available at https://atlas.fredhutch.org/fredhutch/covid/dataset/stephenson. The ulcerative colitis dataset is available through the Single Cell Portal under accession code SCP259. The Perturb-seq data used for generating the simulation dataset is available on Zenodo (https://doi.org/10.5281/zenodo.7041849).
• All original code developed for this study, including for data analysis and model implementation, has been deposited on GitHub and is publicly available at https://github.com/Khreat0205/scMILD.
• Any additional information required to reanalyze the data reported in this paper is available from the lead contact upon request.

## Acknowledgments

The authors thank the anonymous reviewers for their valuable suggestions. This work was funded by the 10.13039/501100003653Korea National Institute of Health via grant (no. 2024-ER-0801-01). The authors thank all members of the lab for their support. The graphical abstract was created with BioRender.com.

## Author contributions

Conceptualization, K.K. and J.C.; methodology, K.J. and K.K.; software, K.J.; validation, K.J.; investigation, K.J.; data curation, K.J.; writing – original draft, J.C.; writing – review and editing, K.K. and J.C.; supervision, K.K. and J.C.

## Declaration of interests

The authors declare no competing interests.

## Declaration of generative AI and AI-assisted technologies in the writing process

During the preparation of this work, the authors used Claude (Anthropic) in order to improve language and readability. After using this tool, the authors reviewed and edited the content as needed and take full responsibility for the content of the publication.

## STAR★Methods

### Key resources table

**Table:** 

REAGENT or RESOURCE	SOURCE	IDENTIFIER
**Deposited data**		

Lupus scRNA-seq Dataset	Mandric et al.^27^	https://github.com/yelabucsf/lupus_1M_cells_clean
COVID-19 Infection scRNA-seq	Ziegler et al.^18^	Single Cell Portal: SCP1289
COVID-19 Hospitalization scRNA-seq	Su et al.^4^; Tian et al.^28^	https://atlas.fredhutch.org/fredhutch/covid/dataset/su
Ulcerative Colitis scRNA-seq	Smillie et al.^6^	Single Cell Portal: SCP259
COVID-19 Longitudinal PBMC	Stephenson et al.^21^; Tian et al.^28^	https://atlas.fredhutch.org/fredhutch/covid/dataset/stephenson
Perturb-seq Data for Simulation	Norman et al.^29^; Peidli et al.^30^	https://doi.org/10.5281/zenodo.7041849

**Software and algorithms**		

scMILD	This study	https://github.com/Khreat0205/scMILD
Hier-MIL	Do et al.^15^	https://github.com/minhchaudo/hier-mil
scPoli	De Dono et al.^16^	https://github.com/theislab/scarches
ProtoCell4P	Xiong et al.^11^	https://github.com/Teddy-XiongGZ/ProtoCell4P
Python version 3.11.14	Python Software Foundation	https://www.python.org
PyTorch version 2.9.0	Pytorch Foundation	https://pytorch.org
R version 4.3.1	R Core Team	https://www.R-project.org
Scanpy version 1.9.6	Wolf et al.^31^	https://scanpy.readthedocs.io
Seurat version 5.0.3	Hao et al.^32^	https://satijalab.org/seurat
Monocle3 version 1.3.7	Cao et al.^33^	https://cole-trapnell-lab.github.io/monocle3/
UCell version 2.8.0	Andreatta et al.^34^	https://github.com/carmonalab/UCell
DESeq2 version 1.42.1	Love et al.^35^	https://github.com/thelovelab/DESeq2
Captum version 0.8.0	Kokhlikyan et al.^36^	https://captum.ai
clusterProfiler version 4.10.1	Yu et al.^37^	https://bioconductor.org/packages/clusterProfiler

### Experimental model and study participant details

This study is purely computational and does not involve direct experimentation on human participants, animals, or cell lines. All single-cell RNA-seq datasets used in this study were obtained from publicly available repositories as detailed in the key resources table. The Lupus dataset comprises 169 samples (50 healthy, 119 SLE patients), the COVID-19 Infection dataset 50 samples (35 COVID-19, 15 normal), the COVID-19 Hospitalization dataset 254 samples (144 hospitalized, 110 mild), the Ulcerative Colitis dataset 30 samples (18 UC patients, 12 healthy individuals), and the COVID-19 Longitudinal dataset 95 samples from 81 patients (12 non-hospitalized, 83 hospitalized), including 27 longitudinal samples from 13 patients. As this study exclusively reanalyzes existing, de-identified datasets, the influence of sex and gender on the results was not independently assessed beyond the original study designs. For detailed demographic information of the study participants, including sex, gender, age, and ancestry, readers are referred to the original publications listed in the key resources table.

### Method details

#### Data acquisition and preprocessing

##### Simulation datasets

To systematically evaluate model performance under controlled settings, we designed simulation studies using preprocessed single-cell RNA sequencing data from a CRISPRa-based gene over-expression experiment.^29^ The data, containing control cells and cells with specific transcription factor perturbations, was preprocessed and filtered to include only cells with good coverage as described previously.^30^ For our primary simulation, we utilized *KLF1*-perturbed cells (1,843 cells) as ground truth condition-specific cells. To simulate more complex scenarios, a mixture simulation dataset was constructed by incorporating both *KLF1*- and *CEBPE*-perturbed cells (1,172 cells). For all simulation datasets, we identified 2,000 highly variable genes (HVGs) for feature selection using scanpy.^31^ The detailed design of the simulation experiments is described in the “Simulation Study Design” subsection below.

##### Disease datasets

We evaluated scMILD on four public single-cell RNA-seq datasets representing various disease conditions. These include a Lupus dataset (169 samples, 834,096 cells),^27^ a COVID-19 nasal swab dataset (Infection; 50 samples, 26,947 cells),^18^ a COVID-19 peripheral blood mononuclear cell (PBMC) dataset (Hospitalization; 254 samples, 515,141 cells),^4^ and an Ulcerative Colitis (UC) dataset (30 samples, 18,725 cells).^6^ All COVID-19 datasets used in this study (Infection, Hospitalization) were obtained from pre-processed data curated previously.^28^ For feature selection, we selected 3,000 HVGs for the Lupus, COVID-19 Infection, and COVID-19 Hospitalization datasets, and 2,000 HVGs for the UC dataset. For the COVID-19 Infection dataset, an additional filter was applied to retain genes expressed in at least five cells. Raw counts were used as model input for all datasets.

##### External validation dataset for temporal analysis

For validation of our sample-informed temporal analysis, we employed an independent longitudinal PBMC dataset from COVID-19 patients.^24^ This dataset contains 95 samples from 81 patients (12 non-hospitalized, 83 hospitalized) and includes 27 longitudinal samples from 13 patients, enabling the validation of temporal dynamics. Data preprocessing for this validation set followed the same HVG selection protocol as our primary COVID-19 Hospitalization dataset.

#### The scMILD framework

scMILD is a weakly supervised deep learning framework based on Multiple Instance Learning (MIL) designed to classify samples and identify condition-associated cell subpopulations from single-cell transcriptomic data. It comprises a shared Encoder and two specialized branches: a Sample Branch and a Cell Branch.

##### Encoder for cellular representation

The encoder, an autoencoder-based neural network, learns a low-dimensional latent representation of each cell’s gene expression. It takes a raw gene count vector $x_{i,j}$ for cell i in sample j and maps it to a latent vector $z_{i,j}=f_{\text{enc}}\left(x_{i,j}\right)$, where $z_{i,j}\in R^{k}$. The decoder part of the autoencoder is used to reconstruct the gene expression profile. The model is trained to minimize the negative log likelihood of a negative binomial (NB) distribution, which is well-suited for sparse, over-dispersed single-cell RNA-seq data. The NB loss is defined as:

(Equation 1) $$L_{NB}=\frac{1}{N}\overset{M}{\underset{j=1}{\sum }}\overset{n_{j}}{\underset{i=1}{\sum }}\overset{G}{\underset{g=1}{\sum }}-\log\text{NB}\left(x_{i,j,g}|{\hat{x}}_{i,j,g},\theta_{i,j,g}\right)$$

where N is the total number of cells, M is the number of samples, $n_{j}$ is the number of cells in sample j, and $\hat{x}$ and θ are the mean and dispersion parameters of the NB distribution output by the decoder. After pretraining, the trained encoder $f_{\text{enc}}$ is used as a shared feature extractor for both the Sample and Cell branches.

##### Attention module for cell weighting

An attention module, adapted froma standard attention-based MIL framework,^12^ is used to compute an attention score $a_{i,j}\in \left[0,1\right]$ for each cell, reflecting its importance for the sample’s classification. The score is calculated from the latent representation $z_{i,j}$ as:

(Equation 2) $$a_{i,j}=\frac{\exp\left(w^{T}\tanh\left(Vz_{i,j}^{T}\right)\right)}{\sum_{l=1}^{n_{j}}\exp\left(w^{T}\tanh\left(Vz_{l,j}^{T}\right)\right)}$$

where V and w are learnable parameters. The attention scores are normalized to sum to 1 within each sample.

##### Sample branch for sample-level classification

The Sample Branch aggregates cellular information to predict a sample-level label. A sample-level feature vector $v_{j}$ is computed by taking an attention-weighted sum of the cell latent representations: $v_{j}=\sum_{i=1}^{n_{j}}a_{i,j}z_{i,j}$. This vector is then fed into a sample classifier $f_{\text{clf}}^{\text{sample}}$ to predict the sample probability ${\hat{y}}_{j}$. The branch is trained by minimizing the standard binary cross-entropy loss $L_{\text{sample}}$ between the predicted probabilities ${\hat{y}}_{j}$and the true sample labels $y_{j}$:

(Equation 3) $$L_{\text{sample}}=-\frac{1}{M}\overset{M}{\underset{j=1}{\sum }}\left(y_{j}\log\left({\hat{y}}_{j}\right)+\left(1-y_{j}\right)\log\left(1-{\hat{y}}_{j}\right)\right)$$

##### Cell branch for cell-state refinement

The Cell Branch aims to refine the cellular representations by performing cell-level predictions. It uses the attention scores from the Sample Branch to generate pseudo-labels for cells: $\tau_{i,j}=a_{i,j}$ if the sample j is positive $\left(y_{j}=1\right)$, and $\tau_{i,j}=0$ if it is negative $\left(y_{j}=0\right)$. A cell classifier $f_{\text{clf}}^{\text{cell}}$ then predicts a cell-level probability ${\hat{\tau }}_{i,j}$ from the latent vector $z_{i,j}$. The training objective for this branch combines two loss functions. First, a weighted binary cross-entropy loss, $L_{\text{WCE}}$, is used to handle the imbalance in pseudo-labels:

(Equation 4) $$\begin{array}{ll} L_{\text{WCE}} & =-\frac{1}{N}\overset{M}{\underset{j=1}{\sum }}\overset{n_{j}}{\underset{i=1}{\sum }}\left(\lambda \left(1-\tau_{i,j}\right)\log\left(1-{\hat{\tau }}_{i,j}\right)\right)\\ & \left(+\left(1-\lambda \right)\tau_{i,j}\log\left({\hat{\tau }}_{i,j}\right)\right) \end{array}$$

where λ is a weight to balance the contribution of positive and negative pseudo-labels. Second, an orthogonal projection loss (OPL), $L_{\text{OPL}}$, is incorporated to enhance the separability of cellular states within positive samples.^38^ The OPL encourages embeddings of cells with high pseudo-labels $\left(C_{\text{high}}\right)$ to be similar to each other, while being orthogonal to embeddings of cells with low pseudo-labels $\left(C_{\text{low}}\right)$. This is achieved by first clustering cells in positive samples into high- and low-attention groups using a Gaussian Mixture Model, and then optimizing the cosine similarity within and between these groups:

(Equation 5) $$L_{\text{OPL}}=\left(1-\frac{1}{\left|P\right|}\underset{c_{i}=c_{j}}{\sum }\langle z_{i},z_{j}\rangle \right)+\left|\frac{1}{\left|N\right|}\underset{c_{i}\neq c_{k}}{\sum }\langle z_{i},z_{k}\rangle \right|$$

where $c_{i}$ represents the cluster label (high or low) for cell i, |P| and |N| are the numbers of same-cluster and different-cluster pairs, respectively, and ⟨⋅,⋅⟩ is the cosine similarity operator. The total loss for the Cell Branch is $L_{\text{cell}}=L_{\text{WCE}}+\gamma L_{\text{OPL}}$, where γ is a hyperparameter.

#### Model training procedure

The training of scMILD is performed in two stages.

##### Stage 1: Pretraining the autoencoder

The autoencoder is first pretrained on all cells from the dataset to learn robust and generalizable cellular representations by minimizing the negative binomial reconstruction loss $\left(L_{NB}\right)$. Key hyperparameters for this stage include the encoder layer dimensions (512, 256, 128), a learning rate of 0.001, and a maximum of 250 epochs with an early stopping patience of 15. The batch size was set to 128.

##### Stage 2: Alternating optimization of sample and cell branches

After pretraining, the main scMILD model is trained by alternating between optimizing the Sample Branch and the Cell Branch.

1. **Sample Branch Optimization**: The parameters of the encoder, attention module, and sample classifier are updated to minimize the sample-level classification loss $\left(L_{\text{sample}}\right)$.
2. **Cell Branch Optimization**: The parameters of the encoder and cell classifier are updated to minimize the combined cell-level loss $\left(L_{\text{cell}}\right)$, using the attention scores from the latest Sample Branch as pseudo-labels.

The encoder learning rate was fixed at 0.001 across all experiments. The attention module hidden dimension was set to 128. The cell branch learning rate was set to 0.001, with hidden dimensions matching those of the sample branch. For the sample branch, the learning rate was set to 0.0001 for most datasets (0.001 for COVID-19 Hospitalization and some simulation conditions), and the hidden dimensions were set to (16, 16) for most datasets ((128, 128) for COVID-19 Hospitalization, (64, 64) for simulation datasets).

The ratio of optimization steps between the sample and cell branches was set to 1 for most experiments, with the exception of COVID-19 Hospitalization where a ratio of 5 was used. The negative class weight (λ) was selected from {0.1, 0.3, 0.5} based on validation performance. The OPL loss weight (γ) was fixed at 1.0 for all datasets except COVID-19 Hospitalization, where γ = 10.0 was used to enhance cell state separation.

Early stopping was implemented by monitoring the validation sample classification loss $\left(L_{\text{sample}}\right)$, with training terminated when no improvement was observed within a patience window of 10–20 epochs depending on the dataset. In practice, convergence was typically observed within 30–40 epochs. The sample branch learning rate was the primary hyperparameter affecting training stability, and a lower sample-to-cell optimization ratio or higher OPL weight (γ) tended to produce more polarized attention scores, facilitating clearer separation between condition-associated and independent cell populations.

Optimization was performed using the Adam optimizer. Complete hyperparameter configurations for all datasets are provided in Table S2.

#### Simulation Study Design

To assess scMILD’s performance under controlled settings, we constructed binary classification datasets from the simulation data. Each dataset comprised two groups of 14 samples each: control samples (containing only control cells) and case samples (containing a mixture of control and perturbed cells). The number of cells per sample was varied (400, 300, 200, 100), and the proportion of perturbed cells in case samples was also varied (20%, 10%, 5%). To evaluate model robustness against annotation errors, we conducted controlled experiments using the single-perturbation simulation dataset (400 cells per sample, 20% perturbation rate). We systematically introduced label noise by randomly flipping the labels of training samples (from control to case or vice versa), with the number of mislabeled samples ranging from 0 to 5 out of 14 total training samples. For each noise level, we performed eight independent runs and measured sample-level AUROC on a clean test set. This design enabled assessment of each model’s ability to maintain performance despite training on partially incorrect labels. For the mixture simulation, case samples contained 10% *KLF1*-perturbed and 10% *CEBPE*-perturbed cells. This design enabled a rigorous evaluation of both sample classification accuracy and cell-level identification capability under diverse conditions.

#### Downstream analysis of scMILD outputs

##### Cell stratification and condition-association grouping

To systematically analyze cell populations based on their relevance to the sample condition, we stratified cells from the test set using the attention scores generated by scMILD’s attention module. For each dataset, we applied a 2-component Gaussian Mixture Model (GMM) to the distribution of attention scores. This partitioned cells into high-attention and low-attention clusters. Based on this, cells from positive-condition samples (e.g., Infected, Inflamed, or Hospitalized) were categorized into two analytical groups:

• **Condition-associated group**: Cells from positive samples belonging to the high-attention GMM cluster.
• **Condition-independent group**: Cells from positive samples belonging to the low-attention GMM cluster.

Cells from negative-condition samples were analyzed separately as a baseline control group. This stratification strategy enabled a focused comparison between cellular states within positive samples.

##### Interpretation of model decisions via Integrated Gradients

To interpret the model’s decision-making process at the gene level, Integrated Gradients (IG) were employed,^39^ an attribution method that assigns importance scores to input features by integrating gradients along a path from a baseline to the input. IG was implemented using the Captum library^36^ with a zero vector as the baseline and 50 integration steps. For each cell, we computed the attribution of each input gene to the cell’s attention score, which represents the model’s assessment of condition-relevance.

The IG analysis was performed on the 3,000 HVGs used for model training, focusing on CD14^+^ monocytes to elucidate the molecular drivers distinguishing the condition-associated (High-scored) group from the remaining cells. Following the protocol established in scDEAL,^40^ we processed the raw importance scores by taking their absolute values to capture the magnitude of each gene’s contribution. These absolute importance scores were then normalized (scanpy.pp.normalize_total) and log-transformed.

To identify Differentially Important Genes (DIGs), we applied the Wilcoxon rank-sum test (via scanpy.tl.rank_genes_groups) to the processed importance scores, comparing the condition-associated group against the remaining cells. Genes with $|\log_{2}\text{FC}|>0.25$, adjusted p-value <0.01, and minimum detection rate >0.1 were defined as DIGs. An identical analysis pipeline and thresholds were applied to gene expression data to identify DEGs for comparison. The statistical significance of the overlap between DIGs and DEGs was assessed using Fisher’s exact test, and the concordance between importance and expression fold-changes across all 3,000 genes was quantified using Pearson and Spearman correlation coefficients.

##### Calculation of known subcluster/subtype signature scores

To validate the biological relevance of our identified cell subpopulations, we calculated gene signature scores for known cell subtypes or subclusters reported in the original studies. For the Lupus dataset, we computed meta-feature scores for ISG^hi^ SLE-expanded subclusters using the top 100 marker genes from a previous SLE study^17^ with the MetaFeature function in Seurat.^32^ For the COVID-19 infection dataset, we used the UCell package^34^ to calculate scores for known subtypes based on their top 5 marker genes as identified previously.^18^

##### Pseudotime analysis

To investigate cellular trajectories in the Ulcerative Colitis dataset, we performed pseudotime analysis using Monocle3^33^ on the WNT2B+ Fos-lo 2 cell population. The trajectory graph was learned using the learn_graph function with default parameters. The root of the trajectory was set to the principal node with the highest proportion of healthy cells or the lowest mean cell attention score.

##### Sample-informed analysis of cellular states

To investigate functional heterogeneity within condition-associated cells while preserving sample context, we developed a sample-informed analytical workflow. This approach was applied to the COVID-19 Hospitalization dataset, focusing on high-attention CD14^+^ monocytes. We first generated a pseudobulk expression profile for these cells for each sample using Seurat’s AggregateExpression function. Unsupervised clustering was then performed on these pseudobulk profiles to identify distinct sample groups. Functional interpretation of these groups was carried out through differential expression and gene ontology enrichment analysis.

### Quantification and statistical analysis

#### Experimental design and model evaluation

All experiments were designed to ensure robust and reproducible evaluation of model performance. Each dataset was systematically partitioned into training (50%), validation (25%), and test (25%) sets. To ensure statistical robustness, we conducted eight independent runs for each experimental setting and implemented early stopping based on validation metrics.

Our comparative analysis employed a hierarchical approach to systematically dissect the contributions of scMILD’s components. We used a standard attention-based MIL framework (ABMIL^12^) as the foundational baseline. Building upon this, ‘scMILD w/o OPL; incorporates our dual-branch architecture but lacks the final Orthogonal Projection Loss, allowing us to isolate the impact of the dual-branch structure. The full scMILD model then adds the OPL. This tiered comparison enables a clear, stepwise evaluation of each of our architectural innovations. To ensure a fair comparison, these models were provided with identical preprocessed data and utilized the same pretrained autoencoder architecture.

For comprehensive benchmarking, we additionally included ProtoCell4P,^11^ Hier-MIL,^15^ and scPoli,^16^ representing recent advances in single-cell-based sample classification. Hyperparameter configurations for scMILD variants are detailed in Table S2. For the additional benchmarked models, we used default settings from their original implementations with the following modifications: Hier-MIL was configured with n_tuning_trials=1 to minimize hyperparameter optimization overhead, while scPoli used the same training parameters reported in its original sample classification analysis, including early stopping. For ProtoCell4P, default hyperparameters were used. To ensure fair comparison, all models were evaluated on identical train/validation/test splits.

#### Performance metrics

Sample-level classification performance was primarily assessed using the area under the receiver operating characteristic curve (AUROC) and the macro-averaged F1-score. For the simulation studies, where ground truth cell labels were available, cell-level identification performance was also measured by the AUROC of cell attention scores against the true cell labels. For the simulation studies, where ground truth cell labels were available, cell-level identification performance was measured by the AUROC of cell attention scores against the true cell labels for models that generate such scores (scMILD, scMILD w/o OPL, and ABMIL). Sample-level performance for all tested models is reported in Table S13.

Clustering performance in the mixture simulation was quantified using the adjusted Rand index (ARI) and adjusted mutual information (AMI). For evaluating the separation of attention score distributions between perturbed and control cells in simulations, Kolmogorov-Smirnov (KS) statistics and overlap coefficients were calculated.

#### Computational scalability analysis

We evaluated computational scalability using the Lupus dataset under two experimental designs. For all scalability experiments, the input data was restricted to the 3,000 highly variable genes (HVGs) used in the main Lupus classification task. For cell-scaling experiments, we systematically downsampled the entire dataset to 50k, 100k, 200k, 400k, and 800k cells (including training, validation, and test sets) while maintaining the original sample composition. For sample-scaling experiments, we randomly selected 20, 50, 80, 110, or 150 samples and downsampled to approximately 200k total cells when necessary, resulting in 95-105k training cells across conditions.

To ensure comparable measurements, early stopping was disabled, and the main training phase for all models was fixed at 100 epochs. For scMILD variants (scMILD, scMILD w/o OPL, ABMIL), we measured the wall-clock time for both Stage 1 (autoencoder pretraining, 250 epochs) and Stage 2 (main training, 100 epochs). ProtoCell4P and scPoli were run with default hyperparameters, except for disabling early stopping. Hier-MIL was configured with n_tuning_trials=1 to minimize hyperparameter optimization overhead.

Computational resources were controlled by limiting each process to 8 CPU threads (via OMP_NUM_THREADS, MKL_NUM_THREADS, OPENBLAS_NUM_THREADS, and torch.set_num_threads(8)). All experiments were performed on a dedicated server (AMD EPYC 7763 64-Core Processor, 1TB RAM, NVIDIA RTX A6000 GPU) to ensure hardware consistency. Due to distinct software dependencies, each model was run in its specific environment, as detailed in Table S17. Time measurements reflect wall-clock execution time, and peak GPU memory was measured using torch.cuda.max_memory_allocated() for all models except Hier-MIL, which required external GPUtil monitoring due to its implementation architecture.

#### Statistical analysis

All statistical analyses were performed using R (v4.3.1) or Python (v3.11.14).

##### Differential gene expression (DEG) analysis

DEGs between specified cell groups were identified using the FindMarkers or FindAllMarkers functions in the Seurat R package,^32^ which implements a Wilcoxon rank-sum test by default. A gene was considered differentially expressed if the Bonferroni-corrected adjusted p-value was less than 0.01 and the absolute log2 fold-change was greater than 0.25, unless otherwise specified. For the sample-informed analysis, DEG analysis on pseudobulk profiles was performed using the DESeq2 package^35^ with an adjusted p-value cutoff of 0.01.

##### Gene ontology (GO) enrichment analysis

Functional enrichment analysis of DEG lists was performed using the clusterProfiler R package.^37^ GO terms in the “Biological Process” ontology were considered significantly enriched if the associated p-value and q-value were less than 0.05.

##### Correlation and association tests

Correlations between variables, such as signature scores and pseudotime, were assessed using Pearson or Spearman correlation coefficients, as specified in the text. Associations between categorical variables, such as sample cluster membership and time points, were evaluated using Fisher’s exact test. For paired comparisons of longitudinal samples, the Wilcoxon signed-rank test was used. For comparisons between two independent groups, the Wilcoxon rank-sum test was used. Statistical significance for all tests was generally defined as p < 0.05, unless otherwise noted.

### Additional resources

This study does not include clinical trials; no clinical registry numbers apply.

## Supplemental information

Document S1. Figures S1–S5

Table S1. Performance comparison between scMILD and the baseline model across eight experimental runs

Table S2. Hyperparameters and model configurations for all experiments

Table S3. Comparison of attention score separability metrics across simulation settings

Table S4. Comparison of DEG identification performance on single-perturbation simulation dataset

Table S5. Comparison of biological relevance of identified clusters in the mixture simulation

Table S6. Top 100 differentially expressed genes (DEGs) for ISGhi SLE-expanded subclusters

Table S7. Cell type counts in the COVID-19 infection test dataset by sample phenotype

Table S8. Distribution of SARS-CoV2 positive cells across subgroups in the COVID-19 infection dataset

Table S9. Differentially expressed genes in Ciliated cells from the COVID-19 infection test dataset

Table S10. Differentially expressed genes in Goblet cells from the COVID-19 infection test dataset

Table S11. Differentially expressed genes in CD14^+^ Monocytes from the COVID-19 Hospitalization test dataset

Table S12. Correlation between CD14^+^ monocyte subtype proportions and clinical variables in SLE

Table S13. Simulation performance across different experimental conditions

Table S14. Robustness to label noise in simulated datasets

Table S15. Computational scalability metrics for cell-scaling experiments

Table S16. Computational scalability metrics for sample-scaling experiments

Table S17. Specifications of the software environment for each model

Table S18. List of Differentially Important Genes (DIGs) and comparison with Differentially Expressed Genes (DEGs) in CD14^+^ Monocytes, including fold-changes and classification categories
