# Supplementary material for: scMILD: Single-cell multiple instance learning for sample classification and associated subpopulation discovery
Source: iScience. 2026 Mar 10;29(4):115284. doi: 10.1016/j.isci.2026.115284 (PMC13019583; doi:10.1016/j.isci.2026.115284)
Supplement: Table S18. List of Differentially Important Genes (DIGs) and comparison with Differentially Expressed Genes (DEGs) in CD14+ Monocytes, including fold-changes and classification categories [file mmc19.pdf]

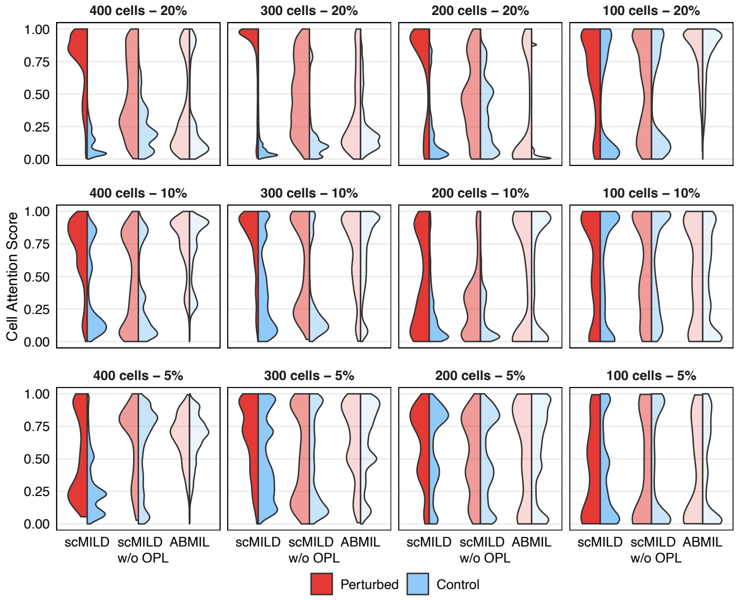

Figure S1: Cell attention score distributions for perturbed and control cells across simulation settings. Split violin plots of cell attention score distributions from scMILD, scMILD w/o OPL, and ABMIL models for perturbed (red) and control (blue) cells across varying total cell numbers (400, 300, 200, and 100 cells per sample).

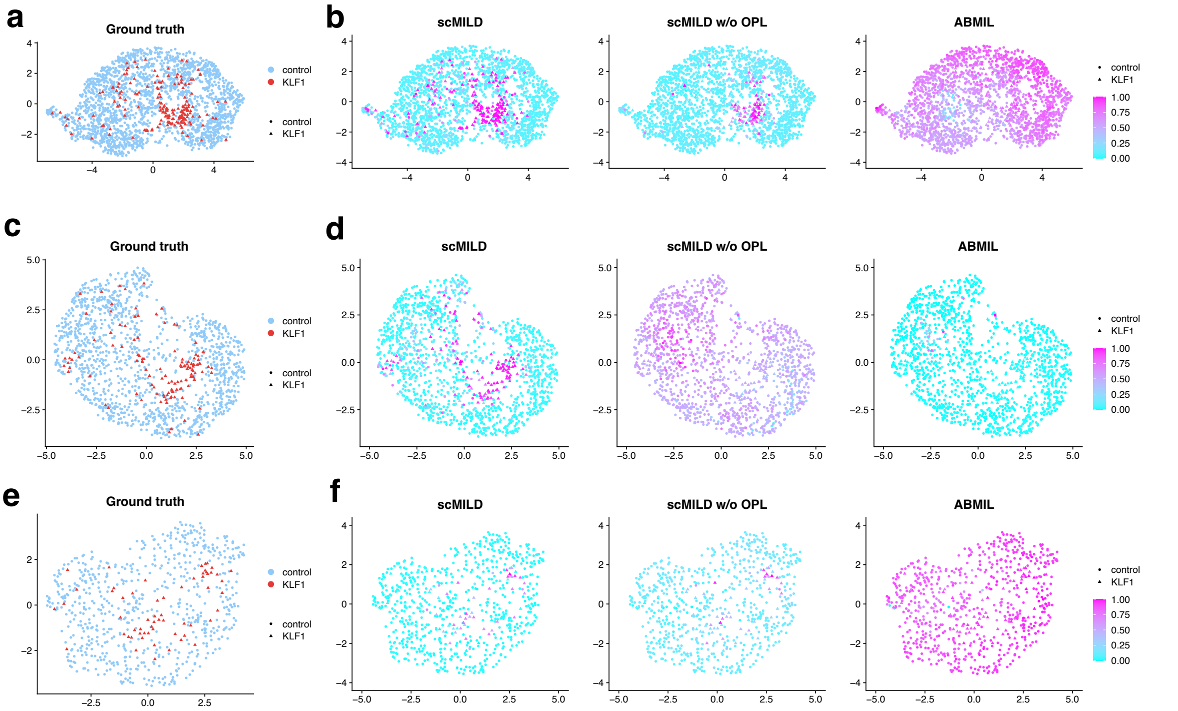

Figure S2: UMAP visualizations of cell labels and attention scores under reduced cell number conditions. a, b.) Experiment with 300 cells per sample. c, d.) With 200 cells per sample. e, f.) With 100 cells per sample, each with 20% perturbed cells. In each set, the left panel shows ground truth cell labels, with perturbed cells represented as red triangles and control cells as blue circles; the right panel shows cell attention scores from scMILD, scMILD w/o OPL, and ABMIL models.

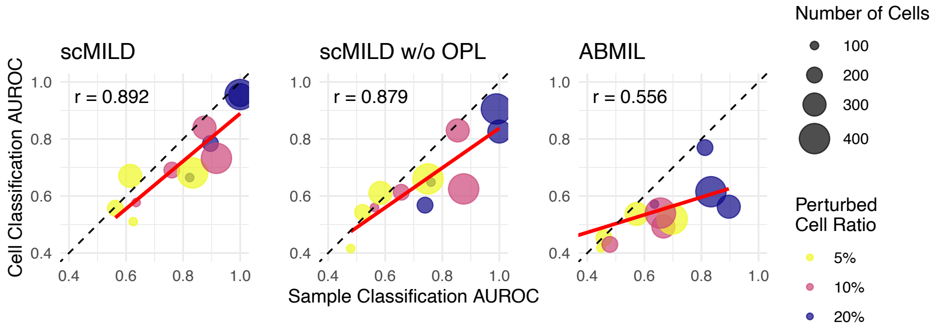

Figure S3: Relationship between sample-level and cell-level classification performance across simulation settings. Scatter plots comparing sample AUROC (x-axis) and cell AUROC (y-axis) for scMILD, scMILD w/o OPL, and ABMIL. Each point represents a simulation setting, with point size indicating the total number of cells per sample and color representing the fraction of perturbed cells.

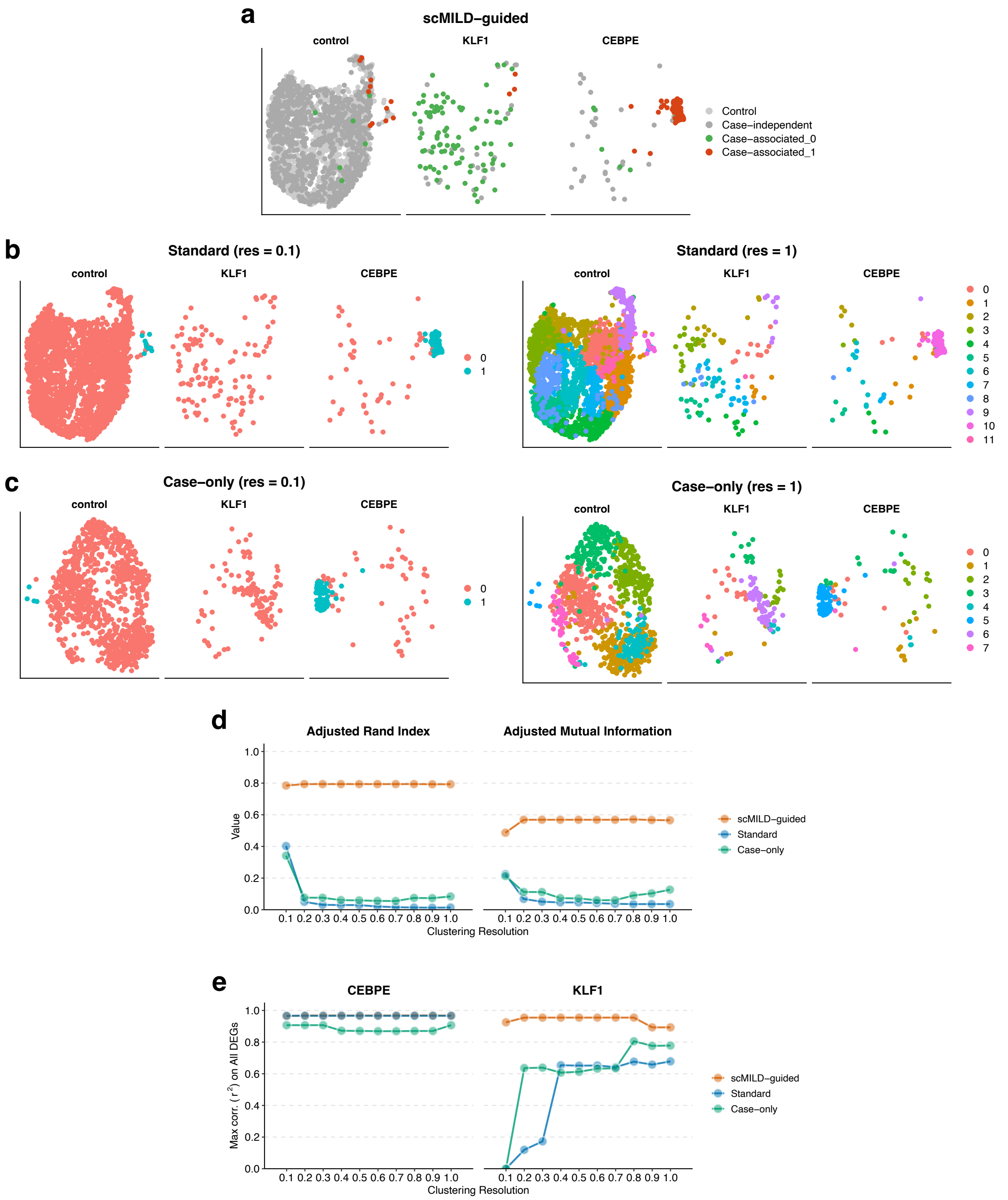

Figure S4: Comparison of clustering strategies and their biological relevance in the mixture simulation. a-c.) UMAP visualizations of clustering results from (a) scMILD-guided clustering, (b) standard clustering (at resolutions 0.1 and 1.0), and (c) case-only clustering (at resolutions 0.1 and 1.0). d.) Line plots showing clustering performance metrics (Adjusted Rand Index and Adjusted Mutual Information) across a range of resolution values for the different clustering strategies. e.) Maximum coefficient of determination (r^2) from Pearson correlation between the log2 fold-change (log2FC) values from cluster-based DEG analysis and ground truth DEG analysis, calculated using all genes and plotted across a range of resolution values.

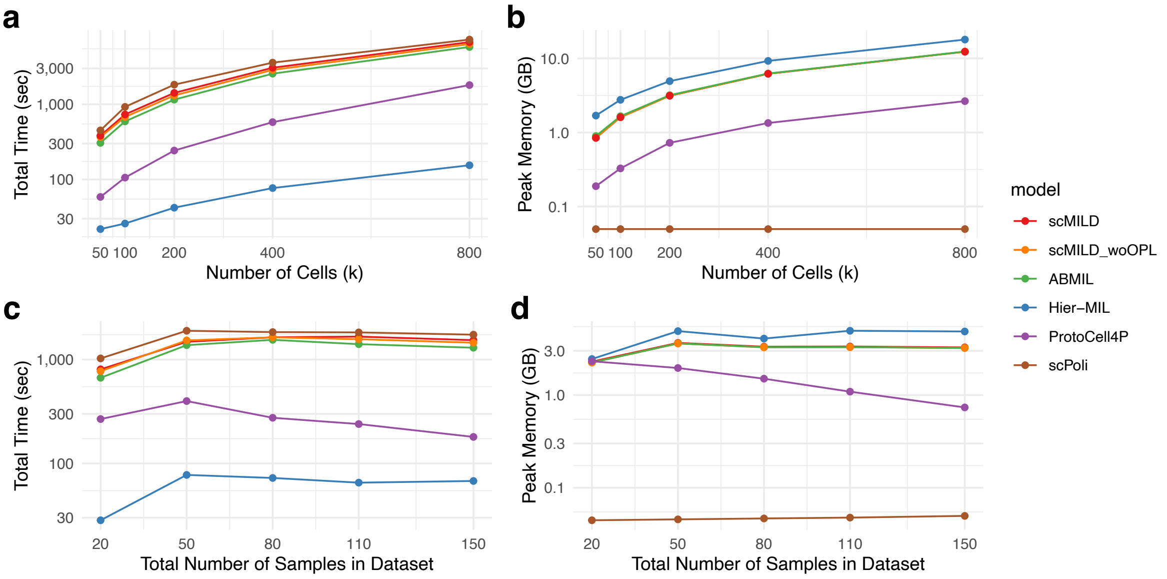

Figure S5: Computational scalability analysis of scMILD and baseline models on the Lupus dataset. a, b.) Line plots showing the total training time (a) and peak GPU memory usage (b) across varying total cell numbers (50k to 800k cells). The dataset was systematically downsampled while maintaining the original sample composition. c, d.) Performance metrics for sample scalability, displaying total training time (c) and peak GPU memory usage (d) across varying numbers of samples (20 to 150 samples). Note that total cell counts were capped at approximately 200k for larger sample sizes, resulting in stabilized resource usage from 50 to 150 samples. In all experiments, models were trained for a fixed 100 epochs without early stopping to ensure fair comparisons.
